# The cGAS–STING pathway in pulmonary infectious and sterile inflammation: differences, connections, and therapeutic implications

**DOI:** 10.3389/fimmu.2026.1867680

**Published:** 2026-08-12

**Authors:** Chenglin Liu, Lannan Yang, Jingjing Yang, Junyan Wang, Jiamin Zhao, Xia Ding, Qian Ni

**Affiliations:** 1 The Second Clinical Medical College, Lanzhou University, Lanzhou, China; 2 Department of Neonatology, The Second Hospital of Lanzhou University, Lanzhou, China; 3 Department of Pediatric Respiratory Medicine, The Second Hospital of Lanzhou University, Lanzhou, China

**Keywords:** biomarkers, cGAS–STING pathway, fibrosis, infectious diseases, pulmonary inflammation, sterile inflammation

## Abstract

The cyclic GMP–AMP synthase–stimulator of interferon genes (cGAS–STING) signaling pathway plays a pivotal role in mediating both infectious and sterile pulmonary inflammation, exhibiting distinct dynamic characteristics under different contexts. The activation kinetics of the cGAS–STING pathway are highly dependent on disease context. In many acute infections, the cGAS–STING pathway is often activated rapidly and induces a strong type I interferon (IFN-I) response, which helps control pathogens. In contrast, chronic sterile injury is usually associated with endogenous damage-associated molecular patterns (DAMPs), persistent low-level pathway activation, inflammatory remodeling, and fibrosis. However, these patterns should be viewed as two ends of a dynamic infectious–sterile continuum rather than as mutually exclusive categories. In many pulmonary diseases, including tuberculosis, chronic viral infection, chronic obstructive pulmonary disease (COPD) exacerbation, infection-associated acute respiratory distress syndrome (ARDS), and post-infectious fibrosis, infectious and sterile mechanisms may coexist, overlap, or occur sequentially. This review summarizes the similarities, differences, and mechanistic connections between cGAS–STING signaling in infectious and sterile lung inflammation. We focus on upstream triggers, signaling dynamics, cell-specific responses, intercellular cyclic GMP-AMP (cGAMP) transmission, inflammatory outcomes, biomarkers, and therapeutic implications. We also propose a temporal–intensity model of cGAS–STING signaling as a conceptual framework to better understand stage-specific pathway functions and support future translational research.

## 1 Introduction

Pulmonary inflammatory diseases exhibit marked heterogeneity across the infectious and sterile spectrum and constitute a major global burden on hospitalization, critical care, and long-term respiratory health (1). Infectious pneumonia encompasses viral, bacterial, and fungal etiologies. Due to factors such as seasonal epidemics, nosocomial infections, and opportunistic infections in immunocompromised populations, there is a significant upward trend in disease-related mortality, chronic pulmonary dysfunction, and long-term care demands (2, 3). Sterile pulmonary inflammation includes ventilator-induced lung injury, ischemia–reperfusion lung injury, radiotherapy- and chemotherapy–induced lung tissue damage, environmental exposures (such as dust inhalation), as well as chronic inflammatory–fibrotic conditions including idiopathic pulmonary fibrosis and chronic obstructive pulmonary disease (4–7). These etiologies often occur in combination and exhibit a disease course characterized by acute exacerbations and chronic progression, leading to impaired oxygenation, prolonged hospitalization, and reduced quality of life (8, 9). With advances in medicine, diagnostic and therapeutic strategies for pulmonary inflammation have made substantial progress in pathogen identification, supportive care, and symptomatic treatment (10). However, effective and standardized evidence-based interventions for severe lung injury and respiratory fibrosis remain lacking. These observations highlight the need for continued mechanistic research and therapeutic strategy development along shared pathological axes to achieve more precise clinical management and drug development.

In the context of diverse etiologies, the cyclic GMP–AMP synthase–stimulator of interferon genes (cGAS–STING) pathway has emerged as a central signaling hub integrating pathogen-associated molecular patterns (PAMPs) and damage-associated molecular patterns (DAMPs). This pathway exerts context-dependent effects spanning host defense and tissue pathology (11, 12). Upon sensing cytosolic double-stranded DNA (dsDNA), cGAS synthesizes the second messenger 2′3′-cGAMP. The latter binds to and activates STING, promoting its translocation, recruitment of TBK1, and phosphorylation of IRF3, thereby initiating signaling cascades that induce type I interferons (IFN-I) and interferon-stimulated genes (ISGs). In parallel, STING can also activate NF-κB signaling, regulate autophagy, and intersect with programmed cell death pathways such as necroptosis and pyroptosis (13–17). As a highly exposed mucosal interface, the lung is subjected to infection, mechanical stretch, oxidative stress, radiation exposure, and cell death, all of which can promote the release of endogenous DNA. These dsDNA molecules can activate the cGAS–STING pathway in a spatiotemporally and cell type–specific manner in alveolar macrophages (AMs), type II alveolar epithelial cells (AEC II), and pulmonary microvascular endothelial cells. This context-dependent activation establishes a delicate balance between pathogen clearance and inflammatory amplification, endothelial injury, and fibrotic remodeling, ultimately shaping the trajectory and outcomes of lung injury (18–21).

Despite extensive recent research on the cGAS–STING pathway, several critical gaps still limit its clinical translation. In clinical settings, infectious and sterile inflammatory processes may coexist, occur sequentially, or evolve into one another. The acute phase driven by pathogens often triggers or amplifies DAMP-mediated chronic low-grade inflammation. This dynamic overlap complicates etiological classification, biomarker interpretation, and therapeutic decision-making. Currently, there is a lack of an operational and validated temporal framework for intervening in cGAS–STING signaling based on disease progression. Early activation may confer benefits for antiviral or anti–intracellular bacterial defense. However, during persistent inflammation or structural remodeling stages, inhibition of sustained cGAS–STING signaling may be more beneficial for limiting tissue injury and fibrotic remodeling. At present, lung-specific biomarkers suitable for stratification remain insufficient. Circulating ISG signatures may not accurately reflect intrapulmonary pathway activity. Integrating cf-mtDNA, 2′3′-cGAMP, and ENPP1 activity in bronchoalveolar lavage fluid with imaging and gas-exchange assessments may provide greater stratification value but still requires further validation (19, 22–24). This review focuses on the similarities, differences, and mechanistic connections of cGAS–STING signaling in pulmonary infectious and sterile inflammation. We first compare upstream triggers and signaling dynamics, then summarize cell-specific responses and intercellular cGAMP transmission within the lung microenvironment and finally integrate inflammatory outcomes along a continuum from acute lung injury to fibrotic remodeling. Based on this framework, we discuss potential biomarker strategies and stage-specific therapeutic considerations for modulating cGAS–STING signaling in future translational research.

## 2 Overview of the cGAS–STING pathway

### 2.1 Overview of the pathway

The cGAS–STING pathway is directly activated by DNA and is not strictly pathogen-specific, distinguishing it from many other innate immune signaling pathways (25). Therefore, double-stranded DNA (dsDNA) from various sources—including DNA viruses, retroviruses, bacteria, phagocytosed dead cells, and self-DNA—can activate the cGAS–STING pathway through direct interaction with cGAS.

The cGAS–STING–TBK1–IRF3 signaling cascade constitutes a central axis of innate immunity. This pathway contributes to both infectious and sterile pulmonary inflammation, but its activation kinetics and downstream outputs vary according to the initiating stimulus. cGAS–STING signaling is initiated when the cytosolic DNA sensor cGAS binds accessible dsDNA. Its N-terminal region contains clusters of positively charged arginine and lysine residues that facilitate electrostatic interactions with the negatively charged phosphate backbone of DNA, thereby promoting DNA binding (14, 26). Importantly, cGAS–DNA recognition is largely length-dependent rather than sequence-specific, with longer DNA fragments (typically longer than approximately 45 base pairs) inducing more efficient activation (27). These cytosolic DNA ligands can arise through multiple routes, including cell lysis, endocytic or phagocytic uptake, and disruption of cellular or organelle membrane integrity (28, 29). Upon DNA binding, cGAS undergoes conformational activation and catalyzes the synthesis of the cyclic dinucleotide 2′3′-cGAMP from adenosine triphosphate (ATP) and guanosine triphosphate (GTP) (26). DNA binding also promotes liquid–liquid phase separation (LLPS), leading to the formation of condensate-like droplets. These phase-separated condensates facilitate cGAS oligomerization and shield bound DNA from degradation by the exonuclease TREX1, thereby enhancing cGAMP production (30, 31). STING is a transmembrane protein located in the endoplasmic reticulum (ER) and contains four transmembrane domains. Once intracellular cGAMP reaches a critical threshold, it binds to STING with high affinity, triggering its translocation to the Golgi apparatus. At the Golgi, STING undergoes palmitoylation at cysteine residues (Cys88 and Cys91), which facilitates the recruitment of TANK-binding kinase 1 (TBK1); this spatial transition enables productive signaling complex assembly and helps restrict activation to the appropriate subcellular compartment (32–34). TBK1 subsequently phosphorylates the C-terminal domain of STING and activates interferon regulatory factor 3 (IRF3). Once activated, IRF3 translocates into the nucleus, where it initiates downstream transcriptional programs leading to type I interferon production. IFN-I subsequently induces the upregulation of a series of ISGs and further promotes the production of pro-inflammatory cytokines (35).

**Figure 1:**
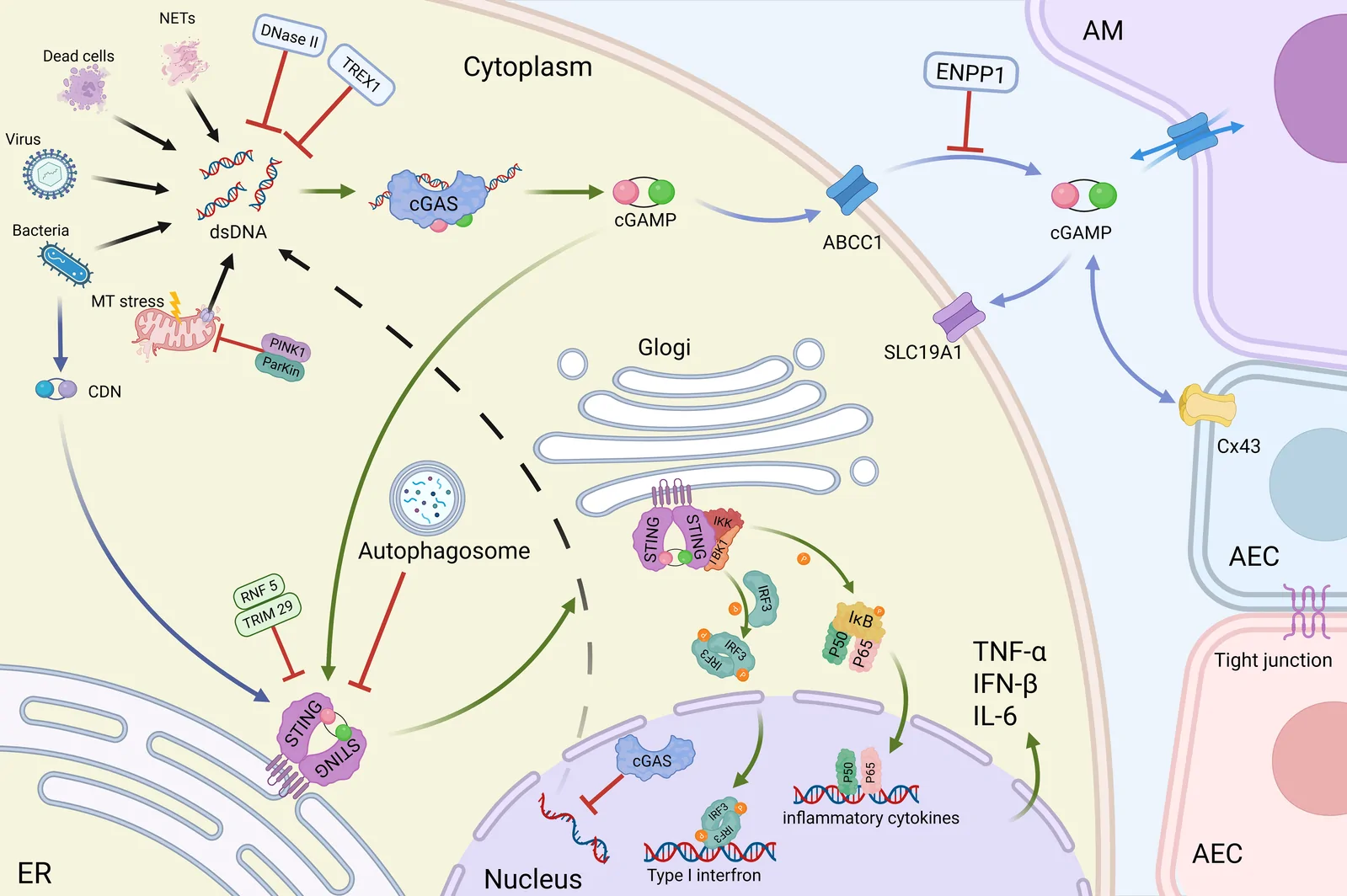
Mechanism of activation, transduction, intercellular communication, and regulation of the cGAS–STING pathway. (1) Induction and Activation: cGAS senses cytosolic dsDNA to produce cGAMP, which triggers STING translocation from the ER to Golgi. This activates TBK1-IRF3 and NF-κB pathways, inducing interferons and pro-inflammatory cytokines. (2) Intercellular Communication: cGAMP can be exported via transporters (ABCC1, SLC19A1) or pass through gap junctions (e.g., Cx43) to reach neighboring AM or AEC, facilitating the spread of immune signals. (3) Multidimensional Regulation: cGAS–STING signaling is tightly regulated by DNA degradation (TREX1, DNase II), mitophagy (PINK1/Parkin), and STING degradation (RNF5, autophagy) to maintain immune homeostasis. Created with BioRender.com.

The NF-κB signaling branch is often activated in parallel, leading to increased expression of inflammatory cytokines and chemokines. However, its activation is generally weaker than that mediated by the Toll/IL-1 receptor domain–containing adaptor-inducing interferon-β (TRIF) and mitochondrial antiviral signaling protein (MAVS) pathways (36, 37). The precise mechanisms underlying STING-dependent NF-κB activation remain incompletely understood. Previous studies have shown that TBK1 undergoes autophosphorylation at Ser172 and subsequently phosphorylates the IKK complex (IKKα/β), which in turn phosphorylates IκBα (the NF-κB inhibitory protein), leading to its ubiquitination and degradation. Degradation of IκBα releases the NF-κB heterodimer (p65 and p50), which, upon activation, translocates to the nucleus and binds κB sites to induce pro-inflammatory genes such as TNF-α, IL-1β, and IL-6 (38, 39). Although the canonical model posits that the C-terminal tail (CTT) of STING is the key structural element for recruiting TBK1 and initiating downstream signaling, other studies demonstrated that STING–NF-κB activation in cellular and murine models does not strictly depend on the CTT. These findings suggest that the mechanisms governing STING–NF-κB activation exhibit structural and functional plasticity (35, 40). Therefore, the mechanisms by which STING activates the NF-κB pathway require further investigation. The cGAS–STING pathway also engages complex molecular mechanisms to induce autophagy. A substantial body of evidence has shown that both STING and cGAS can trigger autophagy through canonical and noncanonical pathways. On the one hand, autophagy facilitates the clearance of cytosolic DNA, damaged mitochondria, and infected organelles, thereby restricting pathogen burden and limiting DAMPs accumulation; on the other hand, it serves as a negative feedback mechanism by targeting excessive or persistently activated STING signaling for lysosomal degradation, thereby constraining signaling duration (41–44) (**Figure 1**).

### 2.2 Intranuclear suppression and micronucleus induction

In addition to the cytosol, cGAS also localizes to the nucleus. Under resting conditions, its binding to the acidic patch of nucleosomes (H2A–H2B) occludes DNA-binding sites and restricts oligomerization, thereby imposing conformational inhibition that prevents aberrant activation of cGAS by genomic DNA (45). In addition, complexes formed by nuclear DNA-binding proteins, such as barrier-to-autointegration factor (BAF) and LEM-domain family proteins at the inner nuclear membrane, preferentially occupy exposed DNA at sites of nuclear envelope stress or rupture. This process contributes to repair while spatiotemporally precluding cGAS access and reducing the likelihood of aberrant activation (46). During mitosis, inhibitory phosphorylation of cGAS by kinases including CDK1 further suppresses its activity. This mechanism serves to minimize the erroneous recognition of self-DNA (47). Consequently, although cGAS is abundant within the cell nucleus, multiple inhibitory mechanisms collectively maintain nuclear cGAS in a largely inactive state under homeostatic conditions. Oxidative stress, radiotherapy, chemotherapeutic agents such as bleomycin, and ventilation-induced lung injury (VILI) can all induce DNA double-strand breaks, nuclear envelope stress, and micronucleus formation. The fragile micronuclear envelope often ruptures under stress, thereby exposing chromatin that is insufficiently shielded by nucleosomes. This process can relieve local constraints on cGAS activation and triggers localized cGAMP synthesis (48–50). Alveolar epithelial cells, particularly AEC II with extensive endoplasmic reticulum and mitochondrial content, are highly sensitive to these stressors and are therefore more prone to activating STING signaling even in the absence of pathogens, resulting in endogenous responses (51, 52).

### 2.3 Negative regulation and DNA clearance

To limit excessive inflammation and tissue damage, pulmonary cells employ multiple layers of negative regulation at both the substrate clearance and signal transduction levels. At the upstream level, the 3′→5′ exonuclease TREX1 rapidly degrades cytosolic DNA, whereas DNase II hydrolyzes DNA derived from both exogenous and endogenous sources (53, 54). Mitochondrial quality control (MQC) promotes mitophagy via the PINK1–Parkin pathway, thereby reducing mtDNA leakage, limiting oxidative modifications, and reducing the upstream supply of cGAS activation at its source (55). At the signaling level, E3 ligases such as RNF5 and TRIM29 promote K48-linked ubiquitination of STING, thereby facilitating its proteasomal degradation or hindering its trafficking to the Golgi apparatus. In contrast, K63-linked polyubiquitination generally supports the assembly and stability of signaling platforms by providing docking sites for effectors containing ubiquitin-binding domains (UBDs). Deubiquitinating enzymes, such as USP21 and USP13 of the USP family, attenuate or terminate downstream signaling by removing K63-linked chains, thereby destabilizing signaling platform assembly (56–59). Extracellularly, membrane-associated or soluble ENPP1 hydrolyzes cGAMP, thereby shortening its half-life and restricting paracrine diffusion, ultimately constraining the spatial boundaries of tissue-level responses (60). Collectively, these negative regulatory mechanisms define the magnitude, duration, and spatial distribution of cGAS–STING signaling.

### 2.4 Intercellular transmission

cGAMP functions both as an intracellular second messenger and as a diffusible “paracrine alarm signal” within lung tissue. Within the alveolar epithelial layer, gap junctions formed by connexins such as Cx43 facilitate lateral diffusion of cGAMP between alveolar epithelial cells. This enables rapid signal synchronization within alveolar units (61, 62). Transmembrane transport of cGAMP is mediated by the reduced folate carrier SLC19A1 and volume-regulated anion channels (VRACs) of the LRRC8 family. These transporters can mediate extracellular cGAMP uptake in several cell types, although their relative contribution in specific pulmonary cell populations remains incompletely defined. Conversely, ABCC1 exports intracellular cGAMP into the alveolar lining fluid or vascular lumen. These transporters together establish local concentration gradients (63–65). Macrophage-derived cGAMP may be transferred to neighboring epithelial or endothelial cells, thereby amplifying antiviral and chemotactic responses. AEC II stimulated by mechanical stress or oxidative stress can also generate cGAMP, thereby activating AMs and endothelial cells. These intercellular coupling and transmembrane transport mechanisms markedly expand the spatial range and functional impact of cGAS–STING signaling at the tissue level.

However, cGAMP propagation is spatially and temporally constrained. ENPP1 possesses extracellular hydrolytic activity, enabling it to rapidly degrade extracellular 2′3′-cGAMP and thereby impose constraints on the spatial gradients and temporal windows of signal transduction (60, 66). The balance between signal propagation and clearance determines whether pulmonary inflammation spreads from focal lesions to a diffuse pattern or remains confined to a local microenvironment. Thus, intercellular cGAMP transmission represents a key mechanism linking local DNA sensing to coordinated tissue-scale immune responses in infectious and sterile lung inflammation.

## 3 Pulmonary inflammation and cGAS–STING signaling

### 3.1 Upstream triggers in pulmonary inflammation

During viral infection, DNA viruses such as adenoviruses and herpesviruses release their genomes into the cytosol during entry or uncoating, where they are directly sensed by cGAS (67, 68). In contrast, RNA viruses, including influenza virus and SARS-CoV-2, lack DNA genomes and therefore activate the cGAS pathway predominantly via indirect mechanisms. First, viral replication and virus protein–mediated cellular stress induce mitochondrial dysfunction and opening of the mitochondrial permeability transition pore (mPTP), resulting in the release of oxidized mtDNA into the cytosol. Second, processes such as cell fusion, nuclear envelope rupture, and micronucleus formation or rupture can expose partially assembled chromatin to the cytosol. Third, under specific infectious conditions, processes such as endogenous reverse transcription or impaired nucleic acid repair can generate DNA intermediates, thereby amplifying the cGAS signaling cascade (19, 69, 70). These DNA signals originate primarily from AEC II and propagate between adjacent epithelial units via cGAMP. This lateral propagation facilitates the rapid and coordinated initiation of IFN-I responses in epithelial cells, effectively priming antiviral defense (19, 28).

Extracellular and facultative pathogenic bacteria can activate the STING pathway through two distinct mechanisms. Most Gram-positive bacteria (such as *Staphylococcus aureus*, *Listeria monocytogenes*, and *Streptococcus pneumoniae*) and a subset of Gram-negative bacteria (such as *Legionella pneumophila* and *Pseudomonas aeruginosa*) are capable of producing cyclic dinucleotides (CDNs), including c-di-AMP, c-di-GMP, and 3′3′-cGAMP. These molecules can directly bind to and activate STING (71, 72). In addition, specific bacterial components (e.g., exotoxins) can induce mitochondrial damage, thereby promoting mtDNA release and the generation of host-derived danger signals (73, 74).

Intracellular bacteria can directly activate cGAS by promoting the exposure of host-derived cytosolic DNA. Previous studies demonstrated that *Mycobacterium tuberculosis* used the ESX-1 (type VII) secretion system to perforate the phagosomal membrane, leading to leakage of both bacterial and host DNA. These DNA molecules induce cGAMP production and activate STING, driving IFN-I expression (75, 76). Notably, IFN-I exhibits pronounced context-dependent effects during tuberculosis infection.

Moderate levels of IFN-I may contribute to limiting bacterial dissemination during the early stages of infection by upregulating ISGs. However, excessive activation of IFN-I is often associated with disease progression. It can inhibit the IL-1 signaling pathway by inducing the IL-1 receptor antagonist (IL-1RA) and perturb the Th1 immune response mediated by the IL-12–IFN-γ axis. Furthermore, IFN-I may also impair host defense and exacerbate tissue damage by affecting the antimicrobial effector functions and autophagic flux of macrophages. Animal model studies showed that the deficiency of cGAS or STING—as well as the blockade of IFNAR1—can significantly improve survival outcomes and attenuate immunopathological damage in lung tissue during tuberculosis infection. However, in certain models, these interventions may be accompanied by a higher bacterial burden during the early stages of infection, suggesting that IFN-I plays a dual role in host anti-tuberculosis immunity that is both time- and context-dependent (77–79). *Legionella* species remodel host membrane systems and perturb mitochondrial homeostasis through Dot/Icm type IV secretion system effectors. These processes can promote the release of mitochondrial DNA and/or exposure of bacterial DNA into the cytosol, which can then be sensed by cGAS and trigger downstream innate immune signaling (80, 81).

In fungal pneumonia, direct evidence implicating cGAS–STING signaling remains limited. Current studies indicate that fungi such as *Candida albicans* and *Aspergillus fumigatus*, following phagocytosis, fungal components and phagolysosomal stress may promote the exposure of fungal DNA or induce host mitochondrial damage, leading to the release of endogenous mtDNA and subsequent activation of cGAS–STING signaling. Fungal virulence factors and reactive oxygen species (ROS) can also promote host mitochondrial damage, thereby leading to mtDNA release and activation of the cGAS–STING pathway (82–84). Experimental evidence further suggests that atypical pathogens such as *Mycoplasma* and *Chlamydia* can indirectly trigger cGAS–STING signaling by inducing DNA damage and micronucleus formation through chronic adhesion and intracellular invasion (85, 86).

Overall, cGAS–STING activation induced by infectious inflammation exhibits three major shared features. First, PAMPs act synergistically with tissue damage–derived signals to drive rapid and robust activation of the cGAS–STING pathway. Second, pathogens can shorten or attenuate signaling duration to achieve immune evasion by encoding cGAMP nucleases (e.g., poxin), promoting ubiquitination and degradation of cGAS/STING, or altering their post-translational modifications (87–89). Third, cGAMP exhibits intercellular transmissibility via ABCC1-mediated export, SLC19A1/LRRC8-mediated uptake, and gap-junction–mediated transfer, while extracellular hydrolysis by ENPP1 imposes spatiotemporal constraints that determine the magnitude and spatial spread of tissue-scale responses (64, 66).

### 3.2 Upstream triggers in sterile lung injury

Sterile lung injury involves diverse types of DAMPs, among which the most prevalent nucleic acids include mtDNA, nuclear DNA (nDNA) fragments, and NET-derived DNA. mtDNA is enriched in unmethylated CpG motifs and lacks nucleosomal protection, rendering it highly susceptible to oxidation and leakage into the cytosol or extracellular space; moreover, its circular structure and bacteria-like features confer strong immunogenicity toward the cGAS–STING and TLR9 pathways (90).

In theory, exposed or partially nucleosome-covered nuclear DNA fragments, which lack nucleosome-mediated inhibition and more readily adopt naked dsDNA conformations that favor cGAS dimerization or phase separation, are more likely to induce cGAS-mediated cGAMP synthesis. However, experimental evidence has shown that leakage of nuclear DNA alone is not sufficient to trigger activation of the cGAS–STING pathway. The intrinsic inhibitory effect of nucleosomes, together with multilayered intracellular regulatory mechanisms—including post-translational modifications of cGAS, restricted binding to the nuclear envelope or nucleosomes, and competitive occupancy by nuclear homeostatic proteins—collectively reduces cGAS sensitivity to nuclear-derived DNA, thereby constraining aberrant activation under homeostatic or noninjurious conditions (45, 91–93). NET-derived DNA originates from neutrophil extracellular traps and contains myeloperoxidase (MPO), neutrophil elastase (NE), and citrullinated histone H3 (CitH3), which enhance immune stimulation and reduce nuclease degradation efficiency, thereby facilitating epithelial uptake of DNA and its entry into the cytosol of neighboring cells (94). In addition, oxidative modifications (such as 8-oxo-dG and base cross-linking) can increase DNA resistance to nucleases and strengthen interactions with pattern-recognition receptors, thereby markedly enhancing immunogenicity, particularly in sterile settings characterized by pronounced oxidative stress (95). These nucleic acid signals display a “low-dose, intermittent” release pattern and often parallel the senescence-associated secretory phenotype (SASP), wherein senescent AEC II, endothelial cells, and fibroblasts continuously generate cytosolic chromatin fragments (CCFs) and sustain low-level IFN-I signaling, thereby establishing a chronic pro-inflammatory microenvironment (96).

In acute sterile lung injury, such as ventilator-induced lung injury (VILI), excessive or repetitive mechanical stretch applied to epithelial and endothelial cells imposes abnormal stress on both the nuclear envelope and mitochondria. High tidal volumes, elevated driving pressures, and increased mechanical power further exacerbate this process: on the one hand, they induce abnormal nuclear envelope tension and micronucleus formation, thereby increasing nuclear DNA (nDNA) leakage; on the other hand, they activate mitochondrial outer membrane permeabilization pathways, promoting the release of oxidized mitochondrial DNA (mtDNA) into the cytosol (50, 97–99). Similarly, A previous study showed that prolonged hyperoxia exposure (≥24–48 hours) leads to sustained accumulation of intracellular reactive oxygen species (ROS) and increased electron leakage from the mitochondrial respiratory chain, thereby creating conditions that may favor recurrent mtDNA-dependent cGAS activation (100). To date, no studies have directly demonstrated that aspiration induces nucleic acid leakage and activates the cGAS–STING pathway. However, acidic injury can trigger lysosomal membrane permeabilization (LMP) and mitochondrial dysfunction; these events have been shown in other tissue injury models to promote cytosolic DNA generation, thereby providing a theoretical basis for potential nucleic acid–sensing mechanisms in aspiration-induced lung injury (101, 102). This remains speculative and requires direct validation in pulmonary aspiration models.

Chronic sterile injury often exhibits relatively consistent features, characterized by repeated or sustained damage to nuclear DNA (nDNA) and mitochondrial DNA (mtDNA), which drives AEC II into senescence and impairs their differentiation capacity. Endogenous DNA released into or accumulated within the cytoplasm activates the cGAS–STING pathway, leading to persistent low-level IFN-I signaling, thereby maintaining or exacerbating cellular dysfunction and promoting tissue fibrosis (96, 103). Agents such as bleomycin and ionizing radiation have been shown to directly induce DNA double-strand breaks, replication stress, increased micronucleus formation, and compromised nucleosome protection, thereby triggering cGAS activation (104, 105).

Environmental pollutants and persistent particulate exposures represent important chronic sterile stimuli in the lung. Unlike acute infectious triggers, these exposures usually induce repeated cellular stress rather than transient inflammatory activation. Continuous oxidative injury, mitochondrial dysfunction, and impaired clearance of endogenous DNA may contribute to persistent cGAS–STING signaling and chronic pulmonary inflammation. Crystalline silica and other mineral particles induce macrophage injury through frustrated phagocytosis and lysosomal destabilization. These processes promote mitochondrial dysfunction, increase mitochondrial reactive oxygen species (mtROS) production, and facilitate mitochondrial DNA (mtDNA) release. The accumulation of cytosolic DNA may activate innate immune pathways, including cGAS–STING signaling, and cooperate with NLRP3 inflammasome activation to amplify sterile inflammation. Similar mechanisms have been proposed for asbestos exposure. However, direct evidence demonstrating sustained cGAS–STING signaling after asbestos exposure remains limited. Asbestos-induced oxidative stress, DNA damage, and chronic macrophage activation may instead create a favorable environment for prolonged DNA sensing and inflammatory amplification.

Chronic exposure to cigarette smoke and PM2.5 induces excessive oxidative stress through reactive metallic and organic components. This stress damages mitochondria and disrupts mitochondrial quality control mechanisms, including PINK1–Parkin-dependent mitophagy. Consequently, damaged mitochondria accumulate and release mtDNA into the cytoplasm, which may sustain cGAS–STING signaling. Environmental particulate exposure also affects alveolar epithelial homeostasis. Cigarette smoke and PM2.5 exposure have been associated with telomere dysfunction, epithelial senescence, and altered differentiation of alveolar epithelial type II cells (AEC II). These changes promote the accumulation of KRT8^+^ transitional epithelial states, which exhibit senescence-associated features and activate profibrotic programs. These transitional cells display SASP-like characteristics, including increased expression of inflammatory and profibrotic mediators such as TGF-β and macrophage-recruiting chemokines including CCL2. These responses contribute to the establishment of a pro-fibrotic microenvironment.

### 3.3 Temporal-intensity dynamics

**Figure 2:**
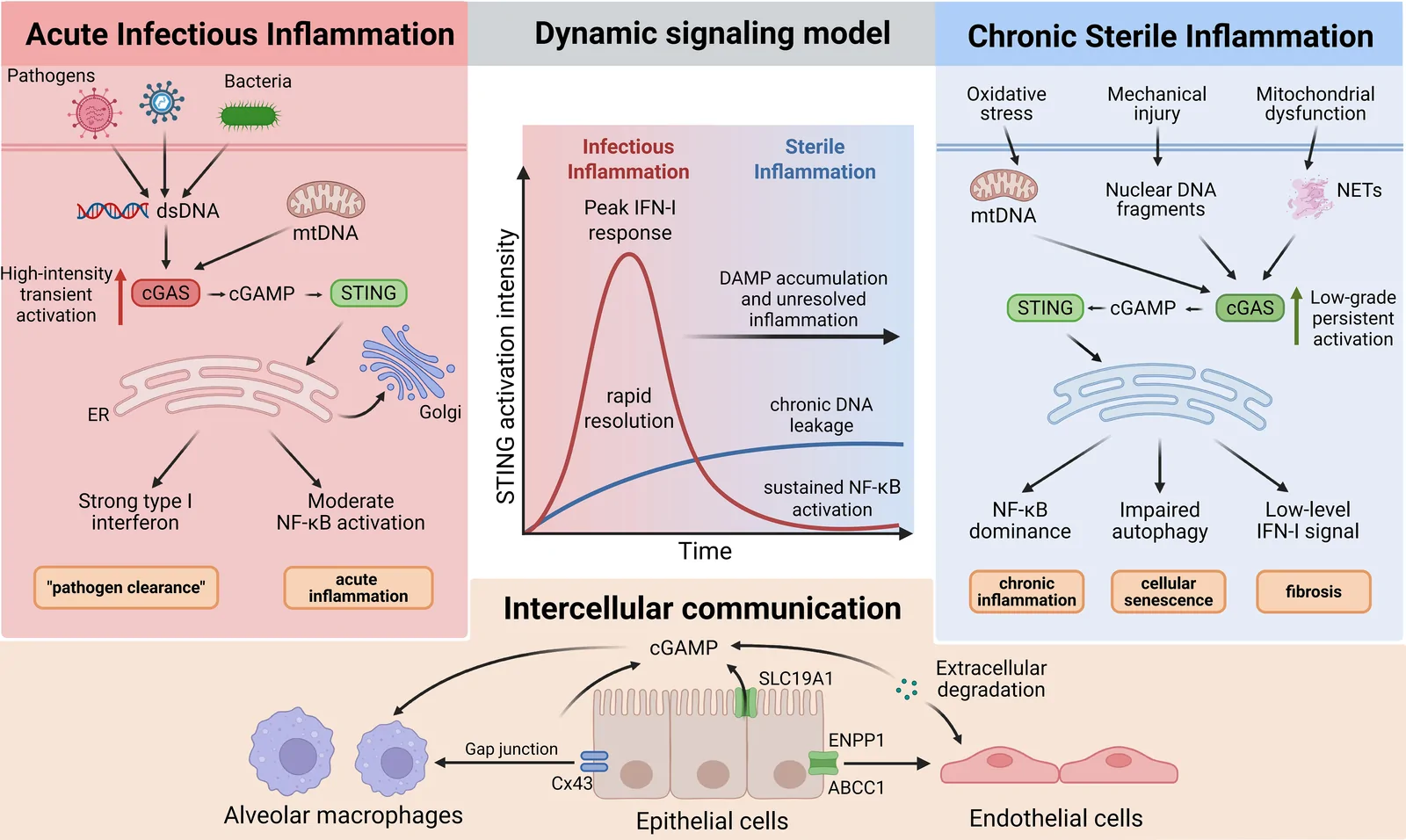
Conceptual temporal–intensity framework of cGAS–STING signaling in infectious and sterile lung inflammation. The schematic depicts spatiotemporal and intensity-dependent regulation of cGAS–STING signaling. (1) In acute infection (left), pathogen-derived DNA rapidly activates the pathway, inducing high but transient signaling with strong IFN-I responses and efficient pathogen clearance. (2) In sterile inflammation (right), persistent release of endogenous DNA sustains low-grade activation, favoring NF-κB signaling, impaired autophagy, and fibrotic remodeling. The central panel illustrates distinct activation kinetics, with a sharp transient peak in infection versus prolonged low-level signaling in sterile conditions, linked by DAMP accumulation and unresolved inflammation. (3) The bottom panel highlights intercellular cGAMP transfer via gap junctions and transporters, with extracellular degradation limiting signal spread. This schematic is intended as a conceptual model illustrating prototypical activation patterns and does not imply that infectious and sterile inflammation are mutually exclusive or fixed categories. Created with BioRender.com.

The biological consequences of cGAS–STING activation are determined not only by the presence of cytosolic DNA but also by the temporal pattern, signaling intensity, and cellular context of pathway activation. These dynamic features are influenced by the origin and persistence of DNA stimuli, the magnitude of cGAMP production, the efficiency of negative feedback mechanisms, and the capacity of cells to restore homeostasis. The temporal–intensity dynamics of cGAS–STING signaling differ across disease contexts, although substantial overlap exists between infectious and sterile inflammation. Multiple studies have suggested that acute infections are often associated with relatively high-intensity, short-duration activation, whereas chronic sterile injury tends to induce lower-intensity but more prolonged activation. Based on these observations, we propose a spatiotemporal and intensity-dependent conceptual framework (**Figure 2**) to summarize the predominant patterns of cGAS–STING signaling in different inflammatory contexts. Importantly, this framework is intended to aid mechanistic interpretation rather than serve as a universal biological rule. Significant overlap can occur between infectious and sterile inflammatory processes in many pulmonary diseases. In infectious inflammation, dsDNA, high concentrations of 2′3′-cGAMP, and bacterial CDNs rapidly induce STING oligomerization, trafficking, and palmitoylation, leading to the formation of high-density signaling microdomains that efficiently recruit TBK1 and activate IRF3, thereby generating robust signaling pulses. These signaling pulses concurrently accelerate STING endocytosis and lysosomal degradation, resulting in rapid signal decay from peak levels within hours and conferring pronounced self-limiting behavior (32, 106, 107). Concurrently, robust IFN-I production induces multiple negative feedback networks—such as USP18-mediated inhibition of IFNAR signaling amplification, USP21/CYLD-mediated removal of K63-linked ubiquitin chains, and TNFAIP3/ABIN1-mediated ubiquitin editing—thereby facilitating rapid post-peak signal attenuation (59, 108, 109). In contrast, sterile stimuli are typically characterized by low-dose, intermittent, and persistent exposure, inducing slower trafficking and palmitoylation of only a subset of STING molecules, thereby prolonging Golgi residence time and reducing peak signaling amplitude. Under this constrained stress-response state, the efficiency of autophagy–lysosome-mediated STING clearance declines, resulting in persistent low-amplitude residual activation. Owing to insufficient early IFN-I production, negative feedback induction remains limited, permitting gradual accumulation driven by chronic senescence-associated secretory phenotype (SASP) signaling and NF-κB activity, ultimately establishing low-amplitude, long-duration signaling states that are tolerated by the host (14, 110, 111). In terms of downstream output profiles, cGAS–STING signaling exhibits context-dependent bias toward IFN-I, NF-κB, and autophagy axes across different cellular lineages and stimulus conditions. In the context of acute infection, cytosolic dsDNA, high concentrations of 2′3′-cGAMP, or bacteria-derived CDNs robustly activate cGAS–STING signaling, driving antimicrobial responses centered on IFN-I and ISGs. In parallel, STING also mediates NF-κB activation; however, this branch primarily functions to upregulate inflammasome-related molecules (e.g., NLRP3 and pro-IL-1β), as well as chemokines and adhesion molecules, thereby promoting immune cell recruitment and the organization of local inflammatory responses. Overall, under acute pathogen challenge, macrophages—particularly AMs—preferentially generate rapid and robust IFN-I bursts via the TBK1–IRF3 axis, whereas NF-κB–dependent inflammatory modules primarily serve auxiliary regulatory roles, enabling the host to establish an effective defensive barrier within a short time frame (39, 112, 113).

Accumulating evidence has shown that persistent endogenous DNA stress induces chronic, low-level activation of cGAS–STING signaling, which is associated with disruption of tissue homeostasis, chronic inflammation, and aging-related phenotypes (111). In addition, mechanistic studies have demonstrated that STING signaling output is not confined to the IRF3–IFN-I axis but is also closely coupled to NF-κB–mediated stress responses, as well as autophagy and lysosomal pathways (114–116). Collectively, these findings suggest that, under sterile or metabolic injury conditions, STING signaling adopts patterns markedly distinct from those observed during acute infection. Integrating the above evidence, we hypothesize that in chronic sterile lung injury, low-dose, repetitive cytosolic DNA stress preferentially induces “subthreshold” or “sustained low-amplitude” STING activation, with downstream outputs biased toward NF-κB and autophagy–lysosomal stress networks rather than robust IRF3–IFN-I responses. Environmental pollutants provide representative examples of this persistent low-amplitude activation pattern. Chronic exposure to cigarette smoke, PM2.5, silica, and other particulate matter induces repeated oxidative stress, mitochondrial dysfunction, and endogenous DNA leakage rather than a single acute inflammatory burst. These processes may continuously supply cytosolic DNA ligands and sustain cGAS–STING signaling over prolonged periods. Therefore, environmental exposure-associated lung injury represents a typical sterile context in which incomplete resolution of cellular stress promotes persistent pathway activity and contributes to epithelial dysfunction, macrophage activation, and fibrotic remodeling. This hypothesis remains to be directly validated in chronic lung disease models and human samples. However, existing primary studies have predominantly focused on acute, high-intensity activation conditions, and direct experimental validation of “chronic–low-intensity–cell lineage–specific” biases in STING activation remains limited. Importantly, this temporal–intensity model should be interpreted as a conceptual framework rather than a universal rule. In clinical practice, infectious and sterile inflammatory mechanisms frequently coexist, overlap, or occur sequentially. For example, tuberculosis and chronic viral infections may maintain persistent pathogen-driven immune activation while simultaneously promoting host-derived DAMPs accumulation. COPD exacerbation often involves infectious triggers superimposed on pre-existing sterile airway inflammation and tissue remodeling. Similarly, infection-associated ARDS and post-infectious fibrosis may evolve from an initial antimicrobial response into a sterile amplification phase characterized by mitochondrial DNA release, NET formation, epithelial injury, endothelial dysfunction, immunothrombosis, and fibrotic remodeling. Therefore, we consider the proposed model a conceptual framework that highlights predominant signaling tendencies along an infectious–sterile continuum, rather than a strict disease classification system.

To help readers evaluate the strength of evidence supporting the proposed framework, we summarized key findings according to disease context, evidence source, and level of support in a Table (**Table 1**). This evidence-grading approach distinguishes relatively established mechanisms from findings supported mainly by animal models, *in vitro* studies, human correlative data, or mechanistic inference. Such distinction is particularly important because the cGAS–STING pathway has been studied across heterogeneous experimental systems, and several aspects of chronic, low-amplitude, cell lineage–specific STING activation in pulmonary inflammation remain incompletely validated.

**Table 1:** Evidence levels for cGAS–STING involvement in pulmonary infectious and sterile inflammation.

Key finding or proposed mechanism	Disease/context	Main evidence source	Interpretation	Evidence strength	Ref.
Cytosolic DNA activates the cGAS–STING pathway	General innate immunity	Biochemical, cellular, and animal studies	Established mechanism	Strong	(12, 26)
DNA viruses directly activate cGAS–STING	DNA viral infections	*In vitro* and animal studies	Established mechanism	Strong	(67, 68)
RNA viruses indirectly activate cGAS–STING via host DNA release	Influenza, SARS-CoV-2	Human, animal, and *in vitro* studies	Context-dependent	Moderate	(19, 70)
Mycobacterium tuberculosis activates cGAS–STING through cytosolic DNA exposure	Tuberculosis	Cell and animal studies	Established, with variable IFN-I effects	Moderate-to-strong	(75, 76)
Bacterial cyclic dinucleotides directly activate STING	Bacterial pneumonia and intracellular infections	Biochemical, *in vitro*, and animal studies	Established mechanism	Strong	(71, 72)
Fungi may activate cGAS–STING through fungal or host DNA	Candida, Aspergillus	Limited *in vitro* and animal studies	Requires further validation	Low-to-moderate	(82–84)
NET-derived DNA activates cGAS–STING and promotes lung injury	ALI, ARDS	Animal and *in vitro* studies	Supported, limited clinical evidence	Moderate	(94, 117)
Sterile lung injury activates cGAS–STING through endogenous DNA	VILI, hyperoxia, radiation injury, IPF	Animal, *in vitro*, and limited human studies	Supported mechanism	Moderate	(18, 50)
Persistent STING activation promotes senescence and fibrosis	IPF, chronic sterile inflammation	Animal, *in vitro*, and human correlative studies	Increasingly supported	Moderate	(96, 103)
Chronic sterile injury may shift STING signaling toward NF-κB and fibrosis	Chronic sterile lung disease	Experimental studies	Proposed mechanism	Low-to-moderate	(114–116)

## 4 Lung microenvironment and cell-specific responses

The preceding sections have delineated the activation mechanisms, signal transduction cascades, and negative regulatory circuits of cGAS–STING signaling. The following sections focus on the pulmonary microenvironmental context and cell-type-specific programs to elucidate their functional specialization and intercellular coupling along the cGAS–STING axis.

### 4.1 Lung microenvironment features

The alveolar–capillary barrier consists of an ultrathin epithelial–basement membrane–endothelial composite structure. While this structure minimizes the diffusion distance, it also markedly increases susceptibility to mechanical and oxidative stress (118). This barrier is covered by surfactant enriched in phospholipids and surfactant proteins. These components not only reduce surface tension but also modulate the membrane lipid microenvironment (119). Studies indicate that exposure to factors such as hyperoxia, cyclic mechanical stretch, and shear stress (e.g., cough- or ventilator-induced lung injury) can induce mitochondrial stress and micronucleus formation. These stressors can increase the likelihood of endogenous DNA leakage (50, 120). Similarly, reperfusion, smoke, particulate matter, and oxidized lipids enhance mitochondrial reactive oxygen species (mtROS) generation and increase the immunogenicity of oxidized DNA, thereby promoting cGAS recognition (118, 121, 122). The alveolar capillary network is highly extensive, and its permeability is tightly regulated. Under inflammatory conditions or protease-mediated injury, disruption of the endothelial glycocalyx compromises barrier integrity and abolishes its anticoagulant properties. This disruption leads to increased vascular permeability and a heightened risk of microthrombosis. These alterations create a favorable environment for the extracellular accumulation and diffusion of DAMPs, as well as the intercellular propagation of cGAS–STING signaling.

### 4.2 Cell-specific roles and crosstalk

AMs represent key effectors of the pulmonary cGAS–STING axis and serve as a major source of IFN-I (predominantly IFN-β) and ISGs. They contribute to pathogen clearance and immune homeostasis under controlled conditions (123). Persistent exposure to pathogen-derived nucleic acids or mitochondrial DNA (mtDNA) can drive sustained STING activation. This leads to metabolic reprogramming, exacerbates inflammatory responses, and disrupts alveolar homeostasis (19). Notably, inflammation-recruited monocyte-derived macrophages exhibit more pronounced pro-inflammatory phenotypes than resident AMs (124). Intercellular transfer of cGAMP between AMs and AEC II enables coordinated amplification of antiviral signaling during early infection. However, when the burden of stimulation exceeds the capacity of autophagy or mitophagy to resolve it, this intercellular propagation becomes dysregulated, resulting in bystander cell injury and amplification of inflammatory cascades (125, 126).

Alveolar epithelial cells (AECs) play complementary roles in maintaining barrier homeostasis and facilitating repair. Type I alveolar epithelial cells (AEC I), which form the ultra-thin gas exchange interface, are highly vulnerable to injury (127). Their disruption compromises barrier integrity and promotes the release of DAMPs, thereby amplifying PRR-dependent inflammatory signaling across multiple cell types (120). In addition, AEC I can sense extracellular cGAMP through transporter-mediated uptake, linking epithelial injury to the propagation of innate immune signaling. Type II alveolar epithelial cells (AEC II) play a central role in alveolar homeostasis, surfactant metabolism, and maintenance of the gas-exchange barrier. Following alveolar injury, they serve as the primary cellular source responsible for cellular replenishment and regeneration, as well as differentiation into AEC I (128). Their high secretory activity renders them particularly sensitive to ER stress and UPR activation. Given that the STING protein is localized to the endoplasmic reticulum, the aforementioned process is mechanistically tightly coupled with mitochondrial dysfunction and the cGAS–STING signaling pathway (129, 130). Moreover, surfactant dysregulation increases alveolar surface tension. This can trigger atelectasis and mechanical stress, leading to increased release of DAMPs (119).

Dendritic cells (DCs) are distributed within subepithelial and interstitial compartments and serve as a critical hub linking innate and adaptive immunity (131). STING activation upregulates IFN-I and costimulatory molecules, enhances cross-presentation, and promotes chemokine production (e.g., CXCL10), thereby bridging pulmonary innate immunity with T cell responses and exerting protective effects in infectious and antitumor contexts (132). In contrast, mature tertiary lymphoid structures (TLS) can persist in chronically inflamed or fibrotic lung tissue, suggesting that sustained low-level inflammatory signaling underlies the maintenance of chronic antigen presentation and local immune cell accumulation (133, 134).

Neutrophils rapidly infiltrate during the acute phase and restrict pathogen dissemination through the formation of neutrophil extracellular traps (NETs). However, NETs are highly immunogenic and can act as DAMPs sensed by cGAS in epithelial and phagocytic cells, thereby triggering STING-dependent mediator production, which in turn promotes neutrophil recruitment and further NET formation, establishing a DNA–NETs–STING amplification loop (94, 117, 135). In addition, NETs directly promote platelet adhesion and activation, as well as fibrin aggregation, leading to endothelial injury and microthrombus formation; subsequent release of DAMPs and complement fragments drives a positive feedback loop linking inflammation, coagulation, and tissue damage (136, 137).

Endothelial cells are highly sensitive to STING signaling. Upon STING activation, pulmonary microvascular endothelial cells exhibit impaired barrier function, upregulated adhesion molecule expression, and an enhanced procoagulant phenotype, thereby promoting leukocyte extravasation and microthrombus formation (138). In addition, complement products (C3a/C5a) and coagulation signaling further potentiate STING and NF-κB pathways, establishing self-amplifying loops of vascular inflammation and coagulation that exacerbate lung injury (139).

In fibroblasts and their matrix microenvironment, STING signaling induces the expression of chemokines such as CXCL10 and multiple extracellular matrix–related genes and, under the coordinated influence of TGF-β signaling, YAP/TAZ activation, and oxidative stress, promotes fibroblast-to-myofibroblast transdifferentiation, accompanied by increased expression and deposition of collagen and fibronectin, thereby accelerating matrix remodeling (140, 141). However, this effect is highly model-dependent, with *in vitro* systems and rodent models providing relatively robust causal evidence, whereas studies in human tissues remain largely correlative.

**Figure 3:**
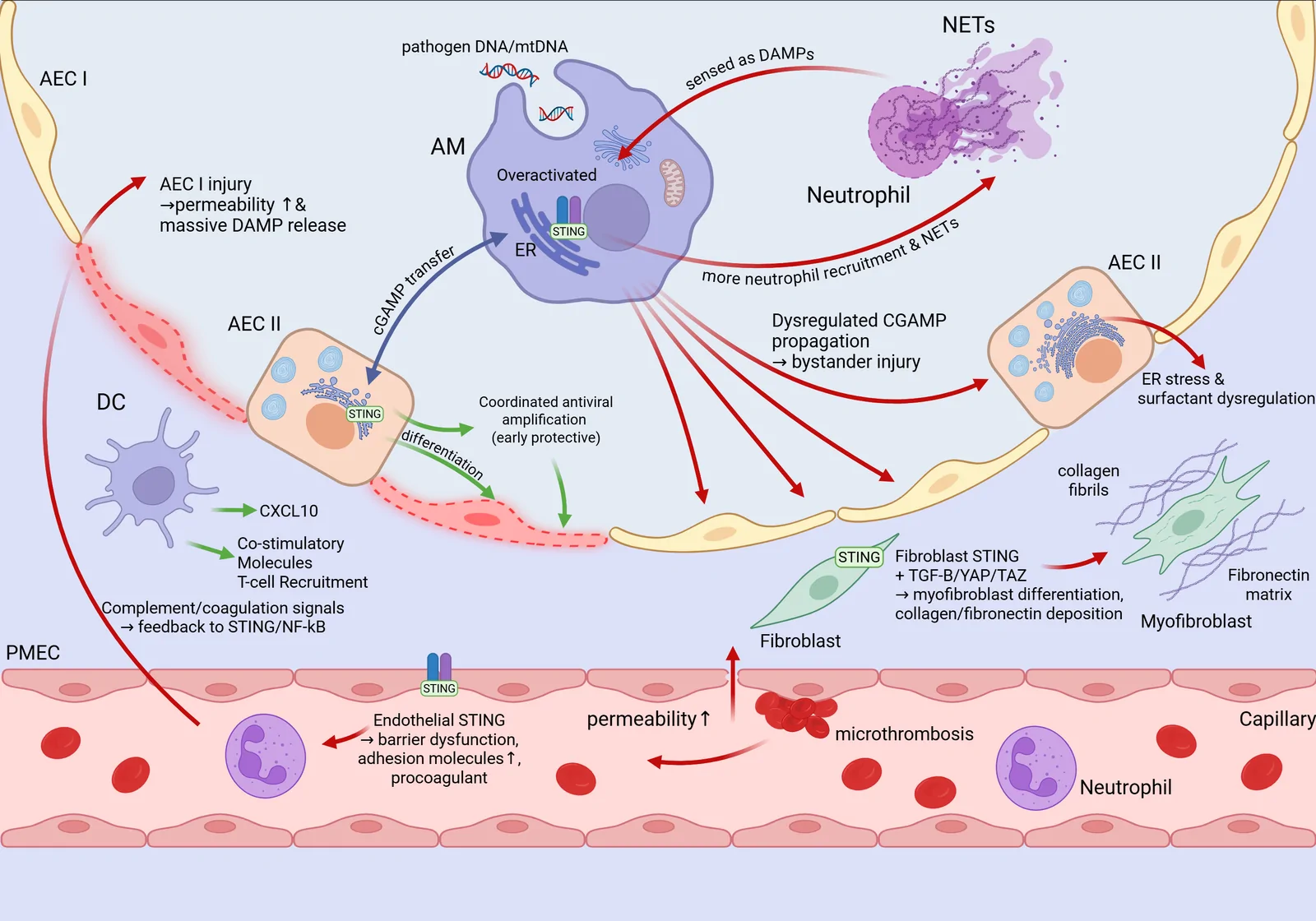
cGAS–STING signaling orchestrates multicellular crosstalk in lung inflammation and fibrosis. Pathogen-derived DNA or mitochondrial DNA activates cGAS–STING signaling in alveolar macrophages (AMs), promoting neutrophil recruitment and NET formation, while amplifying inflammation through cGAMP transfer and DAMP release. Injury to alveolar epithelial cells (AEC I/II) increases permeability and induces ER stress, exacerbating inflammation and bystander damage. Dendritic cells (DCs) enhance antiviral immunity by producing chemokines and co-stimulatory molecules to recruit T cells. Endothelial cGAS–STING activation leads to barrier dysfunction, coagulation activation, and microthrombosis. In fibroblasts, STING cooperates with TGF-β/YAP–TAZ signaling to drive myofibroblast differentiation and extracellular matrix deposition, promoting fibrosis. Overall, the diagram highlights the dynamic balance of the cGAS–STING pathway between host defense and inflammation-driven injury/fibrosis. Created with BioRender.com.

To facilitate cross-cell comparison, the cell type-specific roles of cGAS–STING signaling in the lung microenvironment are summarized in the table (**Table 2**). This table highlights the major activating stimuli, downstream outputs, protective functions, pathological consequences, and evidence strength associated with each pulmonary cell population. Overall, the cGAS–STING axis forms functionally specialized yet interlinked signaling networks across distinct lung cell types, collectively shaping the trajectory from pathogen clearance to immunopathological progression. When signaling is timely and self-limited and intercellular propagation remains coordinated, it favors rapid pathogen clearance and barrier repair; however, once STING signaling across cell populations exceeds a critical threshold, the system shifts into a self-amplifying cycle characterized by increased permeability, inflammatory thrombosis, and matrix remodeling, clinically manifesting as acute respiratory distress syndrome (ARDS) and subsequent post-inflammatory fibrosis (**Figure 3**). Taken together, these cell-specific responses should not be interpreted as independent events. Instead, they form an interconnected pulmonary injury network in which macrophage-derived cGAMP and cytokines amplify epithelial and endothelial activation, epithelial injury releases endogenous DNA and DAMPs, NET-derived DNA further stimulates cGAS–STING signaling, and endothelial dysfunction promotes immunothrombosis. When unresolved, this multicellular amplification loop may ultimately converge on fibroblast activation and extracellular matrix deposition.

**Table 2:** Cell type-specific roles of cGAS–STING signaling in pulmonary inflammation.

Cell type	Main activating signals	Major pathway output	Protective function	Pathological consequence	Ref.
Alveolar macrophages	Pathogen-derived DNA, bacterial CDNs, mtDNA, extracellular cGAMP	IFN-I, ISGs, NF-κB-dependent cytokines and chemokines	Pathogen clearance, antiviral priming, immune coordination	Excessive inflammation, bystander injury, macrophage metabolic dysfunction	(123, 124)
AEC I	Extracellular cGAMP, DAMPs released during barrier injury	STING-dependent inflammatory signaling	Maintenance of gas-exchange barrier integrity	Barrier disruption, DAMP release, amplification of epithelial injury	(120, 127)
AEC II	mtDNA, micronuclear DNA, ER stress, oxidative stress, mechanical stretch	IFN-I, NF-κB, ER-stress-associated signaling	Surfactant homeostasis, epithelial regeneration, antiviral defense	Senescence, impaired repair, profibrotic mediator release	(128)
Dendritic cells	Cytosolic DNA, cGAMP, pathogen-associated signals	IFN-I, CXCL10, antigen-presentation programs	Bridging innate and adaptive immunity, T-cell priming	Chronic antigen presentation, maintenance of tertiary lymphoid structures	(131, 132)
Neutrophils	Infection, DAMPs, inflammatory cytokines	NET formation, inflammatory amplification	Pathogen restriction	NET-derived DNA release, cGAS activation, endothelial injury, immunothrombosis	(94, 117)
Endothelial cells	mtDNA, extracellular cGAMP, complement and coagulation signals	NF-κB activation, adhesion molecules, procoagulant signaling	Vascular immune surveillance, leukocyte recruitment	Barrier leakage, leukocyte extravasation, microthrombosis	(138)
Fibroblasts	Persistent endogenous DNA stress, inflammatory cytokines, TGF-β crosstalk	Chemokines, ECM-related genes, profibrotic signaling	Tissue repair after injury	Myofibroblast differentiation, ECM deposition, fibrosis	(140, 141)

AEC I, type I alveolar epithelial cells; AEC II, type II alveolar epithelial cells; CDNs, cyclic dinucleotides; DAMPs, damage-associated molecular patterns; ECM, extracellular matrix; IFN-I, type I interferon; ISGs, interferon-stimulated genes; mtDNA, mitochondrial DNA; NETs, neutrophil extracellular traps.

## 5 Pulmonary inflammatory outcomes

### 5.1 Infection-driven outcomes

In the context of infectious inflammation, the biological outcomes of the cGAS–STING pathway are determined by the dynamic balance between pathogen clearance and tissue injury. During the early phase of infection, cGAS–STING–mediated IFN-I signaling rapidly induce ISGs and establish an antimicrobial state, thereby suppressing pathogen replication and dissemination (14). Concurrent activation of negative feedback circuits and autophagy–lysosomal pathways promotes inflammatory self-limitation and barrier repair, thereby facilitating restoration of homeostasis (114). However, IFN-I exerts dual effects: while it restricts intracellular pathogens, it can also impair neutrophil and macrophage phagocytic function by suppressing IL-1 signaling, upregulating IL-10, and reshaping chemokine gradients, thereby delaying pathogen clearance and amplifying inflammatory circuits (142). When pathogen burden is high, particularly in combination with ventilation-induced lung injury (VILI) or host susceptibility (e.g., baseline oxidative stress or coagulation abnormalities), excessive or persistent STING activation can induce high-amplitude or delayed IFN-I signaling, thereby reducing epithelial and endothelial metabolic and reparative capacity, increasing cell death, and ultimately driving the development of diffuse alveolar damage (DAD) (19, 143, 144). Histological and physiological features include disruption of epithelial–endothelial junctional complexes, exposure of the basement membrane, reduced alveolar fluid clearance, and consequent development of pulmonary edema and hypoxemia. Plasma protein leakage and fibrin deposition contribute to the formation of hyaline membranes. In addition, endothelial activation, upregulated tissue factor expression, NET formation, and platelet activation collectively promote microthrombus formation (145, 146). These pathological alterations of the alveolar–capillary barrier, together with consolidation and reduced lung compliance, constitute the structural basis of impaired gas exchange.

### 5.2 Sterile injury-driven outcomes

In sterile inflammation, initial injurious stimuli typically drive tissue responses along a staged continuum from “acute injury” to “chronic fibrosis.” During the acute phase, relatively low-intensity but sustained activation of the cGAS–STING pathway, in conjunction with NF-κB and stress–autophagy signaling networks, delays epithelial repair and promotes aberrant regeneration, leading to early exudation and hyaline membrane formation characteristic of diffuse alveolar damage (DAD), thereby predisposing to subsequent fibrosis (147–149). Over time, persistent endoplasmic reticulum (ER) stress and DNA damage induce STING-dependent cellular senescence and the senescence-associated secretory phenotype (SASP), characterized by the release of mediators such as IL-6, TGF-β, and CCL2, which drive the recruitment of monocytes and fibroblast progenitors and promote fibroblast-to-myofibroblast differentiation (20, 150). Integrin αvβ6–mediated activation of latent TGF-β, together with coordinated SMAD signaling and YAP/TAZ-dependent mechanotransduction, further amplifies profibrotic transcriptional programs (140, 151). Excessive deposition of extracellular matrix components, including type I/III collagen and fibronectin, leads to interstitial thickening, impaired gas diffusion, alveolar architectural remodeling, and progressive loss of lung compliance (152). Microvascular endothelial injury, immunothrombosis, and capillary rarefaction contribute to impaired perfusion and chronic hypoxia. In this context, hypoxia-inducible factor (HIF)-driven metabolic reprogramming further exacerbates epithelial–mesenchymal dysregulation (153, 154). Clinical and genetic evidence provide strong support for these mechanisms: STING-associated vasculopathy with onset in infancy (SAVI) manifests as a progressive interstitial lung disease (ILD)-like phenotype driven by constitutive STING activation. Similarly, COPA syndrome results in aberrant STING retention and chronic activation accompanied by pulmonary hemorrhage and ILD-like features (155, 156). Collectively, these observations indicate that, even in the absence of pathogens, sustained cGAS–STING signaling is capable of driving a fibrotic trajectory characterized by epithelial senescence, fibroblast activation, and ECM deposition. This process shifts disease manifestations from acute injury toward persistent impairment of gas exchange and structural remodeling of the lung. Therefore, pulmonary fibrosis can be viewed as one of the major chronic outcomes of sustained sterile cGAS–STING activation. In this setting, the pathway does not merely initiate inflammation but may participate in the transition from epithelial injury and senescence to fibroblast activation, matrix deposition, architectural distortion, and progressive impairment of gas exchange.

### 5.3 The infectious-sterile continuum

In clinical settings, pulmonary inflammation rarely follows a strict separation between infectious and sterile processes. Instead, these conditions frequently represent different phases of a dynamic continuum. During the early stage of infection, cGAS-STING signaling may contributes to host defense by inducing IFN-I production and ISGs, thereby promoting pathogen clearance.However, pathogen elimination does not necessarily terminate cGAS–STING activation. Infection-induced tissue damage can generate secondary sterile signals, including mitochondrial DNA, nuclear DNA fragments, oxidized nucleic acids, and NET-associated extracellular DNA. These endogenous DNA species can sustain cGAS–STING signaling even after pathogen burden decreases, converting an initially protective immune response into a self-amplifying sterile inflammatory program.

This transition is mediated by coordinated responses among multiple pulmonary cell populations. Alveolar macrophages amplify inflammation through continuous sensing and processing of endogenous DNA, whereas injured AEC II undergo stress responses, cellular senescence, and SASP production, thereby promoting epithelial dysfunction and fibrotic remodeling. Endothelial injury further increases vascular permeability and facilitates immune cell recruitment, reinforcing tissue inflammation. Intercellular cGAMP transmission provides an additional mechanism connecting infected and uninfected regions of the lung. Through gap junctions and membrane transporters, cGAMP can propagate STING activation to neighboring cells, extending the inflammatory response beyond the initial site of infection. Therefore, cGAS–STING signaling serves as a molecular bridge linking pathogen recognition, tissue injury, and long-term sterile remodeling.

Given the broad range of pulmonary diseases discussed above, a comparative summary may help clarify how cGAS–STING signaling operates across different pathological contexts. Although these conditions share common features, such as cytosolic DNA sensing, cGAMP-mediated signal propagation, and downstream interferon or inflammatory responses, they differ in the dominant DNA source, main responding cell population, pathway output, pathological consequence, and strength of available evidence. Therefore, we summarized representative disease-specific contexts of cGAS–STING activation in the Table (**Table 3**). This table is intended to provide a structured overview rather than an exhaustive classification of all pulmonary diseases.

**Table 3:** Disease-specific contexts of cGAS–STING activation in pulmonary inflammation.

Disease/context	Major DNA source	Main responding cells	Dominant output	Main outcome	Ref.
Acute DNA viral pneumonia	Viral DNA, host DNA	Epithelial cells, macrophages, DCs	IFN-I, ISGs	Antiviral defense; epithelial injury	(67, 68)
RNA viral pneumonia	mtDNA, micronuclei	Epithelial cells, macrophages, endothelial cells	IFN-I, ISGs, NF-κB	Inflammation, ARDS	(19, 70)
Bacterial pneumonia	Bacterial DNA/CDNs, mtDNA	Macrophages, neutrophils	NF-κB, IFN-I	Pathogen clearance or immunopathology	(71–74)
Tuberculosis	Bacterial DNA, host DNA	Macrophages, DCs, epithelial cells	IFN-I, ISGs, autophagy	Host defense or persistent inflammation	(75, 76)
Fungal pneumonia	Fungal DNA, mtDNA	Macrophages, DCs	IFN-I, cytokines	Antifungal defense or immunopathology	(82–84)
VILI and hyperoxia	mtDNA, nuclear DNA	Epithelial cells, endothelial cells, macrophages	NF-κB, IFN-I	Barrier injury, edema	(50, 100)
Aspiration-induced injury	mtDNA, nuclear DNA, NET DNA	Neutrophils, epithelial cells, macrophages	NF-κB, inflammasome	Acute lung injury, ARDS	(101, 102, 157)
Silica/asbestos exposure	mtDNA, damaged host DNA	Macrophages, epithelial cells, fibroblasts	STING, NLRP3, NF-κB	Inflammation, fibrosis	(158, 159)
Smoke/PM2.5 exposure	Oxidized mtDNA, damaged DNA	Epithelial cells, macrophages	NF-κB, ISGs	Airway inflammation, remodeling	(160, 161)
IPF and therapy-induced fibrosis	mtDNA, micronuclei	AEC II, fibroblasts, macrophages	Senescence, SASP, NF-κB	Fibrosis progression	(162)
COPD exacerbation	Pathogen DNA/CDNs, mtDNA	Epithelial cells, macrophages, neutrophils	NF-κB, IFN-I	Acute exacerbation	(149)
Infection-associated ARDS	Pathogen DNA, mtDNA, NET DNA	Epithelial cells, endothelial cells, macrophages	IFN-I, NF-κB	Diffuse alveolar damage	(19, 143)
Post-infectious fibrosis	DAMPs, mtDNA, NET DNA	AEC II, macrophages, fibroblasts	SASP, NF-κB, TGF-β	Aberrant repair, fibrosis	(144, 152)

## 6 Diagnostic and therapeutic strategies

### 6.1 Biomarkers for cGAS–STING pathway activation

The preceding sections have delineated both the distinctions and interconnections between infectious and sterile pulmonary inflammation at the tissue level. However, translating these mechanistic insights into clinically actionable frameworks requires detectable, reproducible, and biologically interpretable biomarker systems. Within the context of the cGAS–STING pathway, biomarker evaluation may be performed across diverse clinical specimens, including bronchoalveolar lavage fluid (BALF), plasma, sputum, and lung tissue biopsy. These sample types provide complementary information: BALF may better reflect local airway and alveolar immune activity, plasma enables longitudinal monitoring with lower invasiveness, sputum may be useful for airway-dominant inflammation, and tissue samples provide spatial information but are more invasive and less suitable for repeated assessment. Candidate biomarkers can be broadly divided into upstream danger signals, proximal pathway activation markers, downstream interferon/inflammatory outputs, and auxiliary markers of infection or tissue injury. Circulating cell-free mitochondrial DNA (cf-mtDNA), detectable in plasma or BALF by quantitative PCR or digital droplet PCR, reflects mitochondrial injury, cell damage, and DAMPs release. However, it is not specific to cGAS–STING activation and may be affected by sampling procedure, hemolysis, cell contamination, and sample processing (24, 157). In contrast, 2′3′-cGAMP is a more proximal functional readout of cGAS activity and intercellular signal transmission. It can be measured in BALF, plasma, or cell lysates by liquid chromatography–tandem mass spectrometry or immunoassay-based methods, although low abundance, rapid degradation, and limited assay standardization remain important barriers (163). Phosphorylated signaling intermediates, including p-STING, p-TBK1, and p-IRF3, represent core transduction nodes of the pathway. These molecules can be detected in BALF cellular fractions or lung tissue using immunohistochemistry, immunofluorescence, flow cytometry, or western blotting (33, 113). Their specificity for pathway activation is relatively high, but clinical application is limited by the need for cellular or tissue material, antibody variability, and lack of standardized thresholds. ISGs and IFN-β provide downstream evidence of IFN-I pathway activation and can be assessed by qPCR, RNA sequencing, ELISA, or multiplex assays in blood, BALF, or cellular samples (164). However, these markers are not specific to cGAS–STING signaling because other nucleic acid-sensing pathways, including TLR and RLR pathways, may also induce IFN-I responses. Combined assessment of IFN-β with procalcitonin (PCT) and C-reactive protein (CRP) may improve diagnostic specificity by integrating pathway activation with systemic inflammatory status (165, 166). Interleukin-6 (IL-6) and interleukin-1β (IL-1β) serve as broader indicators of downstream inflammatory amplification. These inflammatory markers can be measured in plasma, BALF, or sputum using ELISA or multiplex immunoassays and may reflect inflammatory amplification, cytokine storm, inflammasome activation, or immunothrombotic risk. Nevertheless, they lack specificity for cGAS–STING activation. Soluble triggering receptor expressed on myeloid cells-1 (sTREM-1), commonly measured by ELISA in BALF or serum, may help support infection-associated myeloid activation, especially bacterial pneumonia, but it should not be regarded as a STING-specific biomarker (167, 168). Despite their potential value, several limitations restrict the clinical implementation of cGAS–STING-related biomarkers. First, many biomarkers lack standardized sampling procedures, analytical methods, and diagnostic thresholds. Second, several commonly used indicators, such as IL-6, IL-1β, and IFN-β, are not specific to cGAS–STING signaling. Their levels can also be affected by other inflammatory pathways. Third, direct markers of pathway activation, including cGAMP, p-STING, and p-TBK1, often require complex assays or invasive tissue sampling. These requirements limit their routine clinical use. A composite panel integrating upstream DNA release, proximal pathway activation, downstream interferon and inflammatory outputs, pathogen testing, imaging scores, oxygenation indices, coagulation parameters, and pulmonary function measurements will likely be required for future biomarker-guided stratification. The sample types, detection methods, specificity, limitations, and validation status of representative biomarkers are summarized in the table (**Table 4**).

**Table 4:** Candidate biomarkers for assessing cGAS–STING activity in pulmonary inflammation.

Biomarker	Sample type	Detection method	Main limitations	Current validation status	Ref.
cf-mtDNA	Plasma, BALF	qPCR, ddPCR	Not STING-specific; affected by hemolysis, cell contamination, sampling procedure, and sample processing	Moderately studied in ALI/ARDS, COVID-19, and fibrotic lung disease; not validated as a STING-specific marker	(24, 157)
2′3′-cGAMP	BALF, plasma, cell lysates	LC–MS/MS, ELISA	Low abundance, rapid degradation by ENPP1, limited standardization, uncertain clinical thresholds	Limited; mainly experimental and early translational	(60, 163)
p-STING	BALF cells, lung tissue	IHC, IF, FCM, WB	Requires cellular or tissue material; antibody variability; invasive sampling for tissue analysis	Mostly experimental or tissue-based; limited clinical validation	(32–34)
p-TBK1/p-IRF3	BALF cells, lung tissue	IHC, IF, FCM, WB	Not entirely specific to STING; requires cellular/tissue samples; thresholds not standardized	Mostly preclinical and translational	(35)
ISG signature	Whole blood, plasma cellular fraction, BALF cells, lung tissue	qPCR, RNA-seq, transcriptomic panels	Can be induced by TLR, RLR, and other interferon pathways; influenced by timing and systemic inflammation	Moderately studied in viral infection and interferon-related diseases; not STING-specific	(19, 164)
IFN-β	Plasma, BALF, cell culture supernatant, tissue	ELISA, MIA, qPCR	Low abundance, short half-life, timing-dependent detection, not specific to cGAS–STING	Limited to moderate; context-dependent	(19, 166)
IL-6	Plasma, BALF, sputum	ELISA, MIA, CIA	Broad inflammatory marker; not pathway-specific; influenced by infection, tissue injury, and systemic inflammation	Clinically used as an inflammation marker but not validated for STING activity	(165, 166)
IL-1β	Plasma, BALF, sputum	ELISA, MIA	Low circulating abundance; timing-dependent; not specific to cGAS–STING	Clinically relevant inflammatory marker; limited STING-specific validation	(77, 165)
sTREM-1	BALF, serum/plasma	ELISA, IA	Not related specifically to cGAS–STING; affected by infection type and inflammatory burden	Moderately studied in bacterial pneumonia and sepsis; auxiliary marker only	(167)
ENPP1 activity	BALF, plasma, tissue/cell samples	EAA, LC–MS-based degradation assay	Assays not standardized; unclear pulmonary thresholds; indirect marker of pathway regulation	Low; mainly experimental	(60, 66)

BALF, bronchoalveolar lavage fluid; qPCR, quantitative polymerase chain reaction; ddPCR, digital droplet polymerase chain reaction; LC–MS/MS, liquid chromatography–tandem mass spectrometry; ELISA, enzyme-linked immunosorbent assay; IHC, immunohistochemistry; IF, immunofluorescence; FCM, flow cytometry; WB, western blotting; RNA-seq, RNA sequencing; MIA, multiplex immunoassay; CIA, chemiluminescent immunoassay; IA, immunoassay; EAA, enzymatic activity assay.

### 6.2 Machine learning-assisted biomarker integration

Machine learning-based approaches may offer a useful strategy for integrating cGAS–STING-related biomarkers into disease detection, prognosis, and patient stratification. Recent studies have provided proof-of-concept evidence that cGAS–STING-centered models can extract clinically relevant information from transcriptomic and pathway-level data. Wang et al. developed a transformer-based deep learning model to predict survival based on cGAS–STING pathways. They also built an explainable XGBoost model to predict anti-PD-1/PD-L1 treatment outcomes in hepatocellular carcinoma (169). Similarly, Dai et al. created a cGAS–STING signature for breast cancer. They applied several machine learning methods, including random forest, support vector machine, XGBoost, and neural networks, to predict anti-PD-1 response (170). These studies suggest that cGAS–STING-related features can be used in interpretable models for disease classification and treatment prediction.

Although pulmonary-specific models focusing on cGAS–STING signaling are still limited, similar strategies could be applied to pulmonary infectious and sterile inflammation. Future models may integrate multiple types of data, including cf-mtDNA, 2′3′-cGAMP, phosphorylated STING/TBK1, ISG signatures, cytokine profiles, imaging features, pathogen detection results, oxygenation indices, and clinical parameters. Such multimodal approaches may help distinguish infection-dominant, mixed transitional, and sterile injury-dominant inflammatory states. However, several challenges remain before these approaches can be used in clinical practice. These include the need for prospective validation, testing in external cohorts, improved model interpretability, and standardized methods for biomarker measurement.

### 6.3 Therapeutic modulation of cGAS–STING pathway

**Figure 4:**
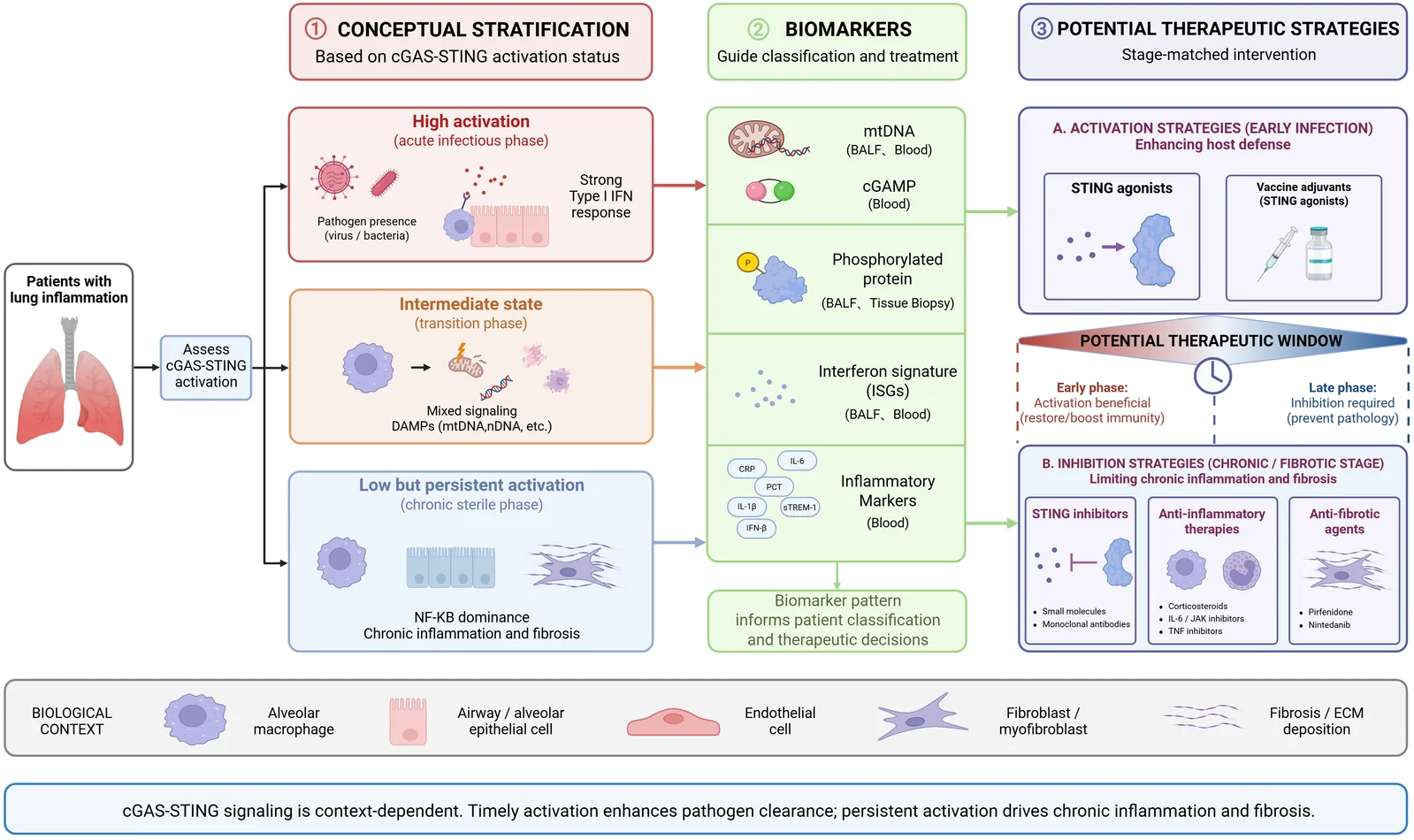
Conceptual translational framework of cGAS–STING signaling in lung inflammation. This schematic presents a hypothesis-generating framework linking cGAS–STING activation patterns with potential biomarker-based stratification and investigational therapeutic directions. The model illustrates three conceptual activation states: acute infection-dominant activation, mixed transitional activation, and chronic sterile injury-dominant activation. Candidate biomarkers, including cGAMP, cf-mtDNA, interferon-stimulated gene signatures, phosphorylated STING/TBK1/IRF3, and inflammatory mediators, may support future disease monitoring after appropriate validation. STING agonism may be explored in selected early viral settings, whereas cGAS–STING inhibition may be investigated in sterile or fibrotic contexts after active infection has been excluded. This framework is conceptual and should not be interpreted as a validated diagnostic algorithm, treatment algorithm, or clinical recommendation. Created with BioRender.com.

Relevant biomarkers may not only support disease subtyping but may also provide longitudinal information for future therapeutic stratification. Building upon the dynamic signaling framework described above, we propose a conceptual translational model that integrates biomarker-based stratification with potential stage-specific therapeutic hypotheses targeting the cGAS–STING pathway (**Figure 4**). This framework should be interpreted as hypothesis-generating rather than as a validated clinical algorithm or treatment recommendation. In settings without evidence of active infection but with features of persistent pathway activation, cGAS–STING inhibition may represent a potential investigational strategy, particularly when accompanied by mitochondrial injury, DAMPs accumulation, or early fibrotic remodeling. Conversely, in selected early viral infections characterized by high viral burden, insufficient antiviral interferon responses, and absence of bacterial or mycobacterial co-infection, localized and short-course STING agonism may be explored as a possible adjunctive approach to enhance mucosal antiviral immunity. However, such strategies remain experimental and require rigorous validation in disease-specific preclinical models and carefully designed clinical studies. Therapeutic decisions targeting the cGAS–STING pathway should not be based on pathway activation alone. Instead, future studies should integrate pathogen detection, longitudinal biomarker reassessment, imaging changes, oxygenation indices, coagulation parameters, and disease stage to evaluate potential benefit and risk. Biomarker reassessment at defined time points, such as 48–72 hours in acute inflammatory settings, may help characterize dynamic changes in pathway activation, but this approach also remains to be prospectively validated.

Therapeutic modulation of the cGAS–STING pathway represents an attractive strategy for pulmonary inflammatory diseases; however, the optimal therapeutic approach depends strongly on disease context. Given the dual functions of cGAS–STING signaling in host defense and inflammatory injury, therapeutic interventions should aim to restore signaling balance rather than simply enhance or suppress pathway activity. In early-stage viral infection characterized by high viral load, insufficient antiviral interferon responses, and no evidence of bacterial pneumonia or mycobacterial infection, pharmacological activation of the cGAS–STING pathway may warrant further investigation as a potential adjunctive approach to induce mucosal IFN-I responses and counteract viral replication. Cyclic dinucleotide (CDN) analogs and their derivatives represent a class of STING agonists with strong immunostimulatory activity. Compared with natural CDNs, synthetic CDNs such as ADU-S100 and MK-1454 exhibit improved stability and have been primarily evaluated in antitumor settings. However, their application in pulmonary inflammatory diseases remains insufficiently explored (171, 172). Small-molecule STING agonists have also attracted attention. For example, amidobenzimidazole-based diABZI compounds have shown antiviral activity in several experimental models, including coronavirus infection models (173). Particular caution is required in bacterial pneumonia, post-viral bacterial superinfection, and tuberculosis. In these conditions, excessive IFN-I signaling may weaken protective antibacterial immunity and delay pathogen clearance. Future studies should establish clear safety monitoring strategies and stopping criteria. Important warning signals may include rapid increases in IL-6 or D-dimer levels, worsening oxygenation, and imaging evidence of diffuse exudation or interstitial edema (174, 175). In contrast, in cases of sterile injury or late-stage infection with a high risk of fibrosis—characterized by elevated ISG levels, increased cf-mtDNA in BALF or plasma, and imaging evidence of diffuse alveolar damage (DAD) or progressive interstitial thickening in the absence of active infection—inhibitory strategies may warrant further investigation. At the pathway level, cGAS antagonists inhibit cGAS activation primarily through two mechanisms: (i) compounds such as RU.365 and RU.521 bind to the catalytic active site and competitively inhibit substrate (ATP or GTP) or product (cGAMP) binding; (ii) agents such as suramin inhibit cGAS activity by targeting the double-stranded DNA (dsDNA) binding interface (176–178). Similarly, STING inhibitors can be classified into two categories: (i) antagonists that competitively occupy the cyclic dinucleotide (CDN) binding site (e.g., Astin C); and (ii) agents that inhibit STING activation by targeting its palmitoylation sites (e.g., C-176). Among these, C-176 selectively binds to STING; studies have demonstrated that pretreatment with C-176 in mice significantly reduces STING agonist–induced increases in serum IFN-I and IL-6 levels (138, 179, 180). In addition, TBK1 inhibitors, such as BX795, can effectively suppress downstream signal transduction. Upstream of the pathway, nucleic acid clearance can be enhanced by promoting autophagy and mitophagy, inhibiting mitochondrial permeability transition pore (mPTP) opening, and regulating DRP1 activity to reduce mitochondrial DNA (mtDNA) release. These approaches may be combined with mitochondria-targeted antioxidants, such as MitoQ, to reduce the supply of DAMPs (181–188). Nevertheless, prolonged pathway inhibition may compromise antimicrobial defense and immune surveillance, particularly in individuals with active infection or increased susceptibility to pathogens. Therefore, future therapeutic strategies should move beyond a simple activation-versus-inhibition paradigm. Integration of disease stage, inflammatory intensity, cellular targets, and biomarker profiles will be essential for developing context-specific approaches to cGAS–STING modulation.

### 6.4 Risks and limitations of therapeutic STING modulation

Despite its therapeutic potential, targeting cGAS–STING signaling remains challenging. The major difficulty is the dual role of this pathway. Transient activation supports host defense and immune surveillance. In contrast, persistent activation may promote chronic inflammation, cellular senescence, and fibrosis. Complete pathway inhibition may also impair protective immune responses. The use of STING agonists requires careful consideration of inflammatory status. In acute infection, enhanced STING activation may improve pathogen clearance; however, in severe pneumonia or post-infectious lung injury, further activation may worsen epithelial damage, endothelial dysfunction, and inflammatory cytokine production. Thus, systemic STING activation may have limited applicability in diseases where excessive inflammation rather than insufficient immunity drives pathology. Similarly, STING inhibition may alleviate chronic sterile inflammation but may also interfere with protective immune responses. This concern is particularly relevant in pulmonary diseases with persistent or recurrent infections, where suppression of DNA sensing pathways could reduce host defense capacity. Therefore, identifying patients with dominant sterile inflammation versus active infection will be critical before clinical translation. Future clinical application of cGAS–STING-targeted therapies will likely require precise patient stratification and dynamic monitoring of pathway activity. Biomarkers reflecting DNA stress, STING activation status, and inflammatory context may help define therapeutic windows and optimize treatment timing.

## 7 Summary and outlook

The cGAS–STING pathway is a central component of the innate immune system. Since cGAS was identified as a cytosolic DNA sensor in 2013, extensive research has progressively elucidated its functional roles and regulatory mechanisms, establishing its importance in antiviral defense, cancer immunity, and autoimmune diseases (12, 14). The functional differences of the cGAS–STING pathway between pulmonary infectious and sterile inflammation can be primarily delineated in three aspects. First, the sources of activation differ: during early infection, activation is primarily driven by PAMPs and pathogen-derived nucleic acids. In contrast, during sterile injury or late-stage infection, activation is predominantly driven by DAMPs, including mitochondrial DNA (mtDNA), micronuclear exposure, and oxidized lipids (28, 72). Second, activation kinetics differ: infectious inflammation is often characterized by high-amplitude, transient pulses of IFN-I, whereas sterile lung injury frequently exhibits relatively low-amplitude but sustained activation (**Figure 2**). Third, the relative contributions of different cell types vary (**Figure 3**): during the acute phase of infection, antiviral clearance is primarily mediated by AMs, conventional type 1 dendritic cells (cDC1), and alveolar epithelial cells (AECs), whereas in the chronic stage, AEC II senescence, endothelial barrier dysfunction, and fibroblast activation become dominant processes (123, 162, 184).

Importantly, the roles of the cGAS–STING pathway in infectious and sterile inflammation are not isolated. Rather, these processes may form a dynamic infectious-sterile continuum, sustained by the persistent replenishment of DAMPs and linked by the intercellular propagation of cGAMP. This continuum implies that even after pathogen burden declines, processes such as neutrophil extracellular trap (NET) formation, pyroptosis, and apoptosis can sustain low-level cGAS–STING signaling. This activation continues to promote progression from diffuse alveolar damage (DAD) to interstitial remodeling and fibrosis (149, 152). Nevertheless, considerable heterogeneity exists among pulmonary diseases and overlap between infectious and sterile mechanisms is common. Therefore, the temporal–intensity framework proposed in this review should be regarded as a conceptual model that captures dominant signaling trends rather than a universally applicable paradigm. Although substantial progress has been made in understanding the cGAS–STING pathway, critical questions regarding its activation, regulation, and *in situ* monitoring in the lung-specific environment remain to be answered. First, during the acute phase, STING signaling supports antiviral defense and contributes to the maintenance of tissue homeostasis. However, this protective role is counterbalanced by epithelial injury, immune–vascular activation, and an increased risk of fibrosis during chronic or dysregulated activation. Notably, different cell lineages exhibit distinct biases toward the IFN-I and NF-κB signaling branches. This observation highlights the need for strategies that enable tunable modulation or branch-selective targeting of the cGAS–STING pathway (19, 148). To address these challenges, future studies should integrate multi-omics profiling with conditional genetic or pharmacologic perturbation approaches to resolve lineage-specific and temporal causal relationships. The development of lung-targeted delivery platforms featuring spatiotemporally controllable release characteristics will be of paramount importance. Such platforms may enable more precise regulation of pathway activity.

Second, cGAMP functions not only intracellularly but also propagates through multiple intercellular pathways within the alveolar microenvironment and is subject to degradation by ENPP1. The thin and structurally complex alveolar interface is likely to influence the effective diffusion length scale and local residence time of cGAMP. This affects the threshold for STING paracrine activation as well as the spatial range of signal propagation (60, 163). Future studies should focus on accurately quantifying the diffusion coefficient of cGAMP in local microenvironments, assessing the impact of ENPP1 activity on its degradation rate, and determining how tethering protein expression levels and transporter distributions influence intercellular transfer. Such insights will facilitate the rational design of local delivery strategies for cGAS–STING agonists or inhibitors, thereby maximizing therapeutic efficacy while limiting paracrine amplification and off-target effects.

Third, precision stratification and adaptive clinical decision-making are essential for the effective therapeutic modulation of the cGAS–STING pathway in pulmonary inflammation. Interventions should be guided by integrated multimodal data—including biomarkers, imaging, and pathogen detection. Such integration may help map patients onto the infectious–sterile continuum and enable time-resolved, phase-specific therapeutic strategies. Future research should prioritize the control of pre-analytical and analytical variability in biomarker measurements, the establishment of standardized assessment criteria, and the development of harmonized diagnostic thresholds. Such standardization may improve inter-center comparability.

This review summarizes the differential roles and intrinsic interconnections of the cGAS–STING signaling pathway in pulmonary infectious and sterile inflammation. Continued investigation into the biphasic effects and temporal dependency of the cGAS–STING signaling across various diseases, with the aim of elucidating the dynamic balance between tissue homeostasis, pathogen clearance, and injury repair, will remain a key long-term research priority. Such efforts will expand the functional scope of the cGAS–STING pathway and provide a useful theoretical foundation for the development and optimization of targeted therapeutic strategies.

## References

[1] Global burden of 369 diseases and injuries in 204 countries and territories, 1990–2019: a systematic analysis for the global burden of disease study 2019. Lancet (Lond Engl). (2020) 396:1204–22. doi: 10.1016/S0140-6736(20)30925-9 33069326 PMC7567026

[2] Dexamethasone in hospitalized patients with covid-19 — preliminary report. N Engl J Med. (2020) 384(8):NEJMoa2021436. doi: 10.1056/NEJMoa2021436 32678530 PMC7383595

[3] Huang L Yao Q Gu X Wang Q Ren L Wang Y . 1-year outcomes in hospital survivors with COVID-19: a longitudinal cohort study. Lancet. (2021) 398:747–58. doi: 10.1016/S0140-6736(21)01755-4 34454673 PMC8389999

[4] Barnes PJ Burney PGJ Silverman EK Celli BR Vestbo J Wedzicha JA . Chronic obstructive pulmonary disease. Nat Rev Dis Primers. (2015) 1:15076. doi: 10.1038/nrdp.2015.76 27189863

[5] Beitler JR Malhotra A Thompson BT . Ventilator-induced lung injury. Clin Chest Med. (2016) 37:633–46. doi: 10.1016/j.ccm.2016.07.004 27842744 PMC5131805

[6] Li Q Nie H . Advances in lung ischemia/reperfusion injury: unraveling the role of innate immunity. Inflammation Res Off J Eur Histamine Res Soc. (2024) 73:393–405. doi: 10.1007/s00011-023-01844-7 38265687

[7] Wang S Xu D Xiao L Liu B Yuan X . Radiation-induced lung injury: from mechanism to prognosis and drug therapy. Radiat Oncol. (2025) 20:39. doi: 10.1186/s13014-025-02617-8 40082925 PMC11907960

[8] Chiumello D Carlesso E Brioni M Cressoni M . Airway driving pressure and lung stress in ARDS patients. Crit Care. (2016) 20:276. doi: 10.1186/s13054-016-1446-7 27545828 PMC4993008

[9] George PM Wells AU Jenkins RG . Pulmonary fibrosis and COVID-19: the potential role for antifibrotic therapy. Lancet Respir Med. (2020) 8:807–15. doi: 10.1016/S2213-2600(20)30225-3 32422178 PMC7228727

[10] Dhanani Z Gupta R . The management of interstitial lung disease in the ICU: a comprehensive review. J Clin Med. (2024) 13:6657. doi: 10.3390/jcm13226657 39597801 PMC11595168

[11] Benmerzoug S Rose S Bounab B Gosset D Duneau L Chenuet P . STING-dependent sensing of self-DNA drives silica-induced lung inflammation. Nat Commun. (2018) 9:5226. doi: 10.1038/s41467-018-07425-1 30523277 PMC6283886

[12] Li XD Wu J Gao D Wang H Sun L Chen ZJ . Pivotal roles of cGAS-cGAMP signaling in antiviral defense and immune adjuvant effects. Science. (2013) 341:1390–4. doi: 10.1126/science.1244040 23989956 PMC3863637

[13] Kelepouras K Saggau J Bonasera D Kiefer C Locci F Rakhsh-Khorshid H . STING induces ZBP1-mediated necroptosis independently of TNFR1 and FADD. Nature. (2025) 647:735–46. doi: 10.1038/s41586-025-09536-4 40834903 PMC12629989

[14] Ablasser A Chen ZJ . cGAS in action: expanding roles in immunity and inflammation. Science. (2019) 363:eaat8657. doi: 10.1126/science.aat8657 30846571

[15] Ablasser A Goldeck M Cavlar T Deimling T Witte G Röhl I . cGAS produces a 2′-5′-linked cyclic dinucleotide second messenger that activates STING. Nature. (2013) 498:380–4. doi: 10.1038/nature12306 23722158 PMC4143541

[16] Burdette DL Monroe KM Sotelo-Troha K Iwig JS Eckert B Hyodo M . STING is a direct innate immune sensor of cyclic-di-GMP. Nature. (2011) 478:515–8. doi: 10.1038/nature10429 21947006 PMC3203314

[17] Gui X Yang H Li T Tan X Shi P Li M . Autophagy induction via STING trafficking is a primordial function of the cGAS pathway. Nature. (2019) 567:262–6. doi: 10.1038/s41586-019-1006-9 30842662 PMC9417302

[18] Huang R Shi Q Zhang S Lin H Han C Qian X . Inhibition of the cGAS-STING pathway attenuates lung ischemia/reperfusion injury via regulating endoplasmic reticulum stress in alveolar epithelial type II cells of rats. J Inflammation Res. (2022) 15:5103–19. doi: 10.2147/JIR.S365970 36091334 PMC9462969

[19] Domizio JD Gulen MF Saidoune F Thacker VV Yatim A Sharma K . The cGAS-STING pathway drives type I IFN immunopathology in COVID-19. Nature. (2022) 603:145–51. doi: 10.1038/s41586-022-04421-w 35045565 PMC8891013

[20] Zhang J Zhang L Chen Y Fang X Li B Mo C . The role of cGAS-STING signaling in pulmonary fibrosis and its therapeutic potential. Front Immunol. (2023) 14:1273248. doi: 10.3389/fimmu.2023.1273248 37965345 PMC10642193

[21] She L Barrera GD Yan L Alanazi HH Brooks EG Dube PH . STING activation in alveolar macrophages and group 2 innate lymphoid cells suppresses IL-33-driven type 2 immunopathology. JCI Insight. (2021) 6:e143509. doi: 10.1172/jci.insight.143509 33400692 PMC7934858

[22] Wang S Böhnert V Joseph AJ Sudaryo V Skariah G Swinderman JT . ENPP1 is an innate immune checkpoint of the anticancer cGAMP-STING pathway in breast cancer. Proc Natl Acad Sci USA. (2023) 120:e2313693120. doi: 10.1073/pnas.2313693120 38117852 PMC10756298

[23] Puray-Chavez M Eschbach JE Xia M LaPak KM Zhou Q Jasuja R . A basally active cGAS-STING pathway limits SARS-CoV-2 replication in a subset of ACE2 positive airway cell models. Nat Commun. (2024) 15:8394. doi: 10.1038/s41467-024-52803-7 39333139 PMC11437049

[24] Fukihara J Sakamoto K Ikeyama Y Furukawa T Teramachi R Kataoka K . Mitochondrial DNA in bronchoalveolar lavage fluid is associated with the prognosis of idiopathic pulmonary fibrosis: a single cohort study. Respir Res. (2024) 25:202. doi: 10.1186/s12931-024-02828-9 38730452 PMC11083749

[25] Ablasser A Hur S . Regulation of cGAS- and RLR-mediated immunity to nucleic acids. Nat Immunol. (2020) 21:17–29. doi: 10.1038/s41590-019-0556-1 31819255

[26] Civril F Deimling T de Oliveira Mann CC Ablasser A Moldt M Witte G . Structural mechanism of cytosolic DNA sensing by cGAS. Nature. (2013) 498:332–7. doi: 10.1038/nature12305 23722159 PMC3768140

[27] Scozzi D Cano M Ma L Zhou D Zhu JH O’Halloran JA . Circulating mitochondrial DNA is an early indicator of severe illness and mortality from COVID-19. JCI Insight. (2021) 6:e143299. doi: 10.1172/jci.insight.143299 33444289 PMC7934921

[28] Kim J Kim HS Chung JH . Molecular mechanisms of mitochondrial DNA release and activation of the cGAS-STING pathway. Exp Mol Med. (2023) 55:510–9. doi: 10.1038/s12276-023-00965-7 36964253 PMC10037406

[29] Gonzales GA Canton J . The delivery of extracellular “danger” signals to cytosolic sensors in phagocytes. Front Immunol. (2022) 13:944142. doi: 10.3389/fimmu.2022.944142 35911757 PMC9329928

[30] W Z L M J M K P . cGAS phase separation inhibits TREX1-mediated DNA degradation and enhances cytosolic DNA sensing. Mol Cell. (2021) 81:742–3. doi: 10.1016/j.molcel.2021.01.024 33606975 PMC7899126

[31] Du M Chen ZJ . DNA-induced liquid phase condensation of cGAS activates innate immune signaling. Sci (N Y NY). (2018) 361:704–9. doi: 10.1126/science.aat1022 29976794 PMC9417938

[32] Mukai K Konno H Akiba T Uemura T Waguri S Kobayashi T . Activation of STING requires palmitoylation at the golgi. Nat Commun. (2016) 7:11932. doi: 10.1038/ncomms11932 27324217 PMC4919521

[33] Shang G Zhang C Chen ZJ Bai X Zhang X . Cryo-EM structures of STING reveal its mechanism of activation by cyclic GMP–AMP. Nature. (2019) 567:389–93. doi: 10.1038/s41586-019-0998-5 30842659 PMC6859894

[34] Hopfner KP Hornung V . Molecular mechanisms and cellular functions of cGAS-STING signalling. Nat Rev Mol Cell Biol. (2020) 21:501–21. doi: 10.1038/s41580-020-0244-x 32424334

[35] Yum S Li M Fang Y Chen ZJ . TBK1 recruitment to STING activates both IRF3 and NF-κB that mediate immune defense against tumors and viral infections. Proc Natl Acad Sci USA. (2021) 118:e2100225118. doi: 10.1073/pnas.2100225118 33785602 PMC8040795

[36] Fang R Jiang Q Zhou X Wang C Guan Y Tao J . MAVS activates TBK1 and IKKϵ through TRAFs in NEMO dependent and independent manner. PLoS Pathog. (2017) 13:e1006720. doi: 10.1371/journal.ppat.1006720 29125880 PMC5699845

[37] Balka KR Louis C Saunders TL Smith AM Calleja DJ D’Silva DB . TBK1 and IKKϵ act redundantly to mediate STING-induced NF-κB responses in myeloid cells. Cell Rep. (2020) 31:107492. doi: 10.1016/j.celrep.2020.03.056 32268090

[38] Ishikawa H Barber GN . STING is an endoplasmic reticulum adaptor that facilitates innate immune signalling. Nature. (2008) 455:674–8. doi: 10.1038/nature07317 18724357 PMC2804933

[39] Abe T Barber GN . Cytosolic-DNA-mediated, STING-dependent proinflammatory gene induction necessitates canonical NF-κB activation through TBK1. J Virol. (2014) 88:5328–41. doi: 10.1128/JVI.00037-14 24600004 PMC4019140

[40] de Oliveira Mann CC Orzalli MH King DS Kagan JC Lee ASY Kranzusch PJ . Modular architecture of the STING C-terminal tail allows interferon and NF-κB signaling adaptation. Cell Rep. (2019) 27:1165–1175.e5. doi: 10.1016/j.celrep.2019.03.098 31018131 PMC7733315

[41] Zhang K Wang S Gou H Zhang J Li C . Crosstalk between autophagy and the cGAS-STING signaling pathway in type I interferon production. Front Cell Dev Biol. (2021) 9:748485. doi: 10.3389/fcell.2021.748485 34926445 PMC8678597

[42] Liang Q Seo GJ Choi YJ Kwak M-J Ge J Rodgers MA . Crosstalk between the cGAS DNA sensor and beclin-1 autophagy protein shapes innate antimicrobial immune responses. Cell Host Microbe. (2014) 15:228–38. doi: 10.1016/j.chom.2014.01.009 24528868 PMC3950946

[43] Prabakaran T Bodda C Krapp C Zhang B-C Christensen MH Sun C . Attenuation of cGAS-STING signaling is mediated by a p62/SQSTM1-dependent autophagy pathway activated by TBK1. EMBO J. (2018) 37:e97858. doi: 10.15252/embj.201797858 29496741 PMC5897779

[44] Liu D Wu H Wang C Li Y Tian H Siraj S . STING directly activates autophagy to tune the innate immune response. Cell Death Differ. (2019) 26:1735–49. doi: 10.1038/s41418-018-0251-z 30568238 PMC6748081

[45] Kujirai T Zierhut C Takizawa Y Kim R Negishi L Uruma N . Structural basis for the inhibition of cGAS by nucleosomes. Science. (2020) 370:455–8. doi: 10.1126/science.abd0237 32912999 PMC7584773

[46] Guey B Wischnewski M Decout A Makasheva K Kaynak M Sakar MS . BAF restricts cGAS on nuclear DNA to prevent innate immune activation. Science. (2020) 369:823–8. doi: 10.1126/science.aaw6421 32792394

[47] Zhong L Hu M-M Bian L-J Liu Y Chen Q Shu H-B . Phosphorylation of cGAS by CDK1 impairs self-DNA sensing in mitosis. Cell Discov. (2020) 6:26. doi: 10.1038/s41421-020-0162-2 32351706 PMC7186227

[48] Durante M Formenti SC . Radiation-induced chromosomal aberrations and immunotherapy: micronuclei, cytosolic DNA, and interferon-production pathway. Front Oncol. (2018) 8:192. doi: 10.3389/fonc.2018.00192 29911071 PMC5992419

[49] Mackenzie KJ Carroll P Martin C-A Murina O Fluteau A Simpson DJ . cGAS surveillance of micronuclei links genome instability to innate immunity. Nature. (2017) 548:461–5. doi: 10.1038/nature23449 28738408 PMC5870830

[50] Lin JY Jing R Lin F Ge WY Dai HJ Pan L . High tidal volume induces mitochondria damage and releases mitochondrial DNA to aggravate the ventilator-induced lung injury. Front Immunol. (2018) 9:1477. doi: 10.3389/fimmu.2018.01477 30018615 PMC6037891

[51] Chung K-P Hsu C-L Fan L-C Huang Z Bhatia D Chen Y-J . Mitofusins regulate lipid metabolism to mediate the development of lung fibrosis. Nat Commun. (2019) 10:3390. doi: 10.1038/s41467-019-11327-1 31358769 PMC6662701

[52] Bueno M Lai Y-C Romero Y Brands J St Croix CM Kamga C . PINK1 deficiency impairs mitochondrial homeostasis and promotes lung fibrosis. J Clin Invest. (2015) 125:521–38. doi: 10.1172/JCI74942 25562319 PMC4319413

[53] Lan YY Londoño D Bouley R Rooney MS Hacohen N . Dnase2a deficiency uncovers lysosomal clearance of damaged nuclear DNA via autophagy. Cell Rep. (2014) 9:180–92. doi: 10.1016/j.celrep.2014.08.074 25284779 PMC4555847

[54] Li T Yum S Wu J Li M Deng Y Sun L . cGAS activation in classical dendritic cells causes autoimmunity in TREX1-deficient mice. Proc Natl Acad Sci USA. (2024) 121:e2411747121. doi: 10.1073/pnas.2411747121 39254994 PMC11420187

[55] Zhou X Wang J Yu L Qiao G Qin D Yuen-Kwan Law B . Mitophagy and cGAS-STING crosstalk in neuroinflammation. Acta Pharm Sin B. (2024) 14:3327–61. doi: 10.1016/j.apsb.2024.05.012 39220869 PMC11365416

[56] Sun H Zhang Q Jing Y-Y Zhang M Wang H-Y Cai Z . USP13 negatively regulates antiviral responses by deubiquitinating STING. Nat Commun. (2017) 8:15534. doi: 10.1038/ncomms15534 28534493 PMC5457515

[57] Li Q Lin L Tong Y Liu Y Mou J Wang X . TRIM29 negatively controls antiviral immune response through targeting STING for degradation. Cell Discov. (2018) 4:13. doi: 10.1038/s41421-018-0010-9 29581886 PMC5859251

[58] Ge J Zhang L . RNF5: inhibiting antiviral immunity and shaping virus life cycle. Front Immunol. (2023) 14:1324516. doi: 10.3389/fimmu.2023.1324516 38250078 PMC10796512

[59] Chen Y Wang L Jin J Luan Y Chen C Li Y . p38 inhibition provides anti-DNA virus immunity by regulation of USP21 phosphorylation and STING activation. J Exp Med. (2017) 214:991–1010. doi: 10.1084/jem.20161387 28254948 PMC5379979

[60] Carozza JA Cordova AF Brown JA AlSaif Y Böhnert V Cao X . ENPP1’s regulation of extracellular cGAMP is a ubiquitous mechanism of attenuating STING signaling. Proc Natl Acad Sci USA. (2022) 119:e2119189119. doi: 10.1073/pnas.2119189119 35588451 PMC9173814

[61] Pépin G De Nardo D Rootes CL Ullah TR Al-Asmari SS Balka KR . Connexin-dependent transfer of cGAMP to phagocytes modulates antiviral responses. mBio. (2020) 11:e03187–19. doi: 10.1128/mBio.03187-19 31992625 PMC6989113

[62] Ablasser A Schmid-Burgk JL Hemmerling I Horvath GL Schmidt T Latz E . Cell intrinsic immunity spreads to bystander cells via the intercellular transfer of cGAMP. Nature. (2013) 503:530–4. doi: 10.1038/nature12640 24077100 PMC4142317

[63] Maltbaek JH Cambier S Snyder JM Stetson DB . ABCC1 transporter exports the immunostimulatory cyclic dinucleotide cGAMP. Immunity. (2022) 55:1799–1812.e4. doi: 10.1016/j.immuni.2022.08.006 36070769 PMC9561016

[64] Ritchie C Cordova AF Hess GT Bassik MC Li L . SLC19A1 is an importer of the immunotransmitter cGAMP. Mol Cell. (2019) 75:372–381.e5. doi: 10.1016/j.molcel.2019.05.006 31126740 PMC6711396

[65] Lahey LJ Mardjuki RE Wen X Hess GT Ritchie C Carozza JA . LRRC8A:C/E heteromeric channels are ubiquitous transporters of cGAMP. Mol Cell. (2020) 80:578–591.e5. doi: 10.1016/j.molcel.2020.10.021 33171122 PMC12629894

[66] Guan D Fang L Feng M Guo S Xie L Chen C . Ecto-nucleotide pyrophosphatase/phosphodiesterase 1 inhibitors: research progress and prospects. Eur J Med Chem. (2024) 267:116211. doi: 10.1016/j.ejmech.2024.116211 38359537

[67] Lam E Stein S Falck-Pedersen E . Adenovirus detection by the cGAS/STING/TBK1 DNA sensing cascade. J Virol. (2014) 88:974–81. doi: 10.1128/JVI.02702-13 24198409 PMC3911663

[68] Reinert LS Lopušná K Winther H Sun C Thomsen MK Nandakumar R . Sensing of HSV-1 by the cGAS-STING pathway in microglia orchestrates antiviral defence in the CNS. Nat Commun. (2016) 7:13348. doi: 10.1038/ncomms13348 27830700 PMC5109551

[69] Cai X Chiu YH Chen ZJ . The cGAS-cGAMP-STING pathway of cytosolic DNA sensing and signaling. Mol Cell. (2014) 54:289–96. doi: 10.1016/j.molcel.2014.03.040 24766893

[70] Moriyama M Koshiba T Ichinohe T . Influenza a virus M2 protein triggers mitochondrial DNA-mediated antiviral immune responses. Nat Commun. (2019) 10:4624. doi: 10.1038/s41467-019-12632-5 31604929 PMC6789137

[71] Lu Z Fu Y Zhou X Du H Chen Q . Cyclic dinucleotides mediate bacterial immunity by dinucleotide cyclase in vibrio. Front Microbiol. (2022) 13:1065945. doi: 10.3389/fmicb.2022.1065945 36619988 PMC9813507

[72] Marinho FV Benmerzoug S Oliveira SC Ryffel B Quesniaux VFJ . The emerging roles of STING in bacterial infections. Trends Microbiol. (2017) 25:906–18. doi: 10.1016/j.tim.2017.05.008 28625530 PMC5650497

[73] Liu N Pang X Zhang H Ji P . The cGAS-STING pathway in bacterial infection and bacterial immunity. Front Immunol. (2021) 12:814709. doi: 10.3389/fimmu.2021.814709 35095914 PMC8793285

[74] Nerlich A Mieth M Letsiou E Fatykhova D Zscheppang K Imai-Matsushima A . Pneumolysin induced mitochondrial dysfunction leads to release of mitochondrial DNA. Sci Rep. (2018) 8:182. doi: 10.1038/s41598-017-18468-7 29317705 PMC5760655

[75] Watson RO Bell SL MacDuff DA Kimmey JM Diner EJ Olivas J . The cytosolic sensor cGAS detects mycobacterium tuberculosis DNA to induce type I interferons and activate autophagy. Cell Host Microbe. (2015) 17:811–9. doi: 10.1016/j.chom.2015.05.004 26048136 PMC4466081

[76] Wassermann R Gulen MF Sala C Perin SG Lou Y Rybniker J . Mycobacterium tuberculosis differentially activates cGAS- and inflammasome-dependent intracellular immune responses through ESX-1. Cell Host Microbe. (2015) 17:799–810. doi: 10.1016/j.chom.2015.05.003 26048138

[77] Mayer-Barber KD Andrade BB Barber DL Hieny S Feng CG Caspar P . Innate and adaptive interferons suppress IL-1α and IL-1β production by distinct pulmonary myeloid subsets during mycobacterium tuberculosis infection. Immunity. (2011) 35:1023–34. doi: 10.1016/j.immuni.2011.12.002 22195750 PMC3246221

[78] Donovan ML Schultz TE Duke TJ Blumenthal A . Type I interferons in the pathogenesis of tuberculosis: molecular drivers and immunological consequences. Front Immunol. (2017) 8:1633. doi: 10.3389/fimmu.2017.01633 29230217 PMC5711827

[79] Moreira-Teixeira L Mayer-Barber K Sher A O’Garra A . Type I interferons in tuberculosis: foe and occasionally friend. J Exp Med. (2018) 215:1273–85. doi: 10.1084/jem.20180325 29666166 PMC5940272

[80] Escoll P Song O-R Viana F Steiner B Lagache T Olivo-Marin J-C . Legionella pneumophila modulates mitochondrial dynamics to trigger metabolic repurposing of infected macrophages. Cell Host Microbe. (2017) 22:302–316.e7. doi: 10.1016/j.chom.2017.07.020 28867389

[81] Ruiz-Moreno JS Hamann L Shah JA Verbon A Mockenhaupt FP Puzianowska-Kuznicka M . The common HAQ STING variant impairs cGAS-dependent antibacterial responses and is associated with susceptibility to legionnaires’ disease in humans. PLoS Pathog. (2018) 14:e1006829. doi: 10.1371/journal.ppat.1006829 29298342 PMC5770077

[82] Marques E Kramer R Ryan DG . Multifaceted mitochondria in innate immunity. NPJ Metab Health Dis. (2024) 2:6. doi: 10.1038/s44324-024-00008-3 38812744 PMC11129950

[83] Brown Harding H Kwaku GN Reardon CM Khan NS Zamith-Miranda D Zarnowski R . Candida albicans extracellular vesicles trigger type I IFN signalling via cGAS and STING. Nat Microbiol. (2024) 9:95–107. doi: 10.1038/s41564-023-01546-0 38168615 PMC10959075

[84] Chen T Feng Y Sun W Zhao G Wu H Cheng X . The nucleotide receptor STING translocates to the phagosomes to negatively regulate anti-fungal immunity. Immunity. (2023) 56:1727–1742.e6. doi: 10.1016/j.immuni.2023.06.002 37379835

[85] Sun G Xu X Wang Y Shen X Chen Z Yang J . Mycoplasma pneumoniae infection induces reactive oxygen species and DNA damage in A549 human lung carcinoma cells. Infect Immun. (2008) 76:4405–13. doi: 10.1128/IAI.00575-08 18663006 PMC2546820

[86] Yang H Sun P Zhou S Tang Y Li S Li W . Chlamydia psittaci infection induces IFN-I and IL-1β through the cGAS-STING-IRF3/NLRP3 pathway via mitochondrial oxidative stress in human macrophages. Vet Microbiol. (2024) 299:110292. doi: 10.1016/j.vetmic.2024.110292 39581075

[87] Yu XF Wang S Ye R Wei W . Viral evasion of cGAS-STING pathway: opportunities for intervention. Trends Pharmacol Sci. (2025) 46:989–1003. doi: 10.1016/j.tips.2025.08.009 40947349

[88] Eaglesham JB McCarty KL Kranzusch PJ . Structures of diverse poxin cGAMP nucleases reveal a widespread role for cGAS-STING evasion in host-pathogen conflict. eLife. (2020) 9:e59753. doi: 10.7554/eLife.59753 33191912 PMC7688311

[89] Yang N Wang Y Dai P Li T Zierhut C Tan A . Vaccinia E5 is a major inhibitor of the DNA sensor cGAS. Nat Commun. (2023) 14:2898. doi: 10.1038/s41467-023-38514-5 37217469 PMC10201048

[90] Giordano L Ware SA Lagranha CJ Kaufman BA . Mitochondrial DNA signals driving immune responses: why, how, where? Cell Commun Signal CCS. (2025) 23:192. doi: 10.1186/s12964-025-02042-0 40264103 PMC12012978

[91] de Oliveira Mann CC Hopfner KP . Nuclear cGAS: guard or prisoner? EMBO J. (2021) 40:e108293. doi: 10.15252/embj.2021108293 34250619 PMC8365253

[92] Zhao B Xu P Rowlett CM Jing T Shinde O Lei Y . The molecular basis of tight nuclear tethering and inactivation of cGAS. Nature. (2020) 587:673–7. doi: 10.1038/s41586-020-2749-z 32911481 PMC7704945

[93] Takaki T Millar R Hiley CT Boulton SJ . Micronuclei induced by radiation, replication stress, or chromosome segregation errors do not activate cGAS-STING. Mol Cell. (2024) 84:2203–2213.e5. doi: 10.1016/j.molcel.2024.04.017 38749421

[94] Zhao J Zhen N Zhou Q Lou J Cui W Zhang G . NETs promote inflammatory injury by activating cGAS-STING pathway in acute lung injury. Int J Mol Sci. (2023) 24:5125. doi: 10.3390/ijms24065125 36982193 PMC10049640

[95] Yoshida H Nishikawa M Kiyota T Toyota H Takakura Y . Increase in CpG DNA-induced inflammatory responses by DNA oxidation in macrophages and mice. Free Radical Biol Med. (2011) 51:424–31. doi: 10.1016/j.freeradbiomed.2011.04.035 21571065

[96] Dou Z Ghosh K Vizioli MG Zhu J Sen P Wangensteen KJ . Cytoplasmic chromatin triggers inflammation in senescence and cancer. Nature. (2017) 550:402–6. doi: 10.1038/nature24050 28976970 PMC5850938

[97] Riley JS Quarato G Cloix C Lopez J O’Prey J Pearson M . Mitochondrial inner membrane permeabilisation enables mtDNA release during apoptosis. EMBO J. (2018) 37:e99238. doi: 10.15252/embj.201899238 30049712 PMC6120664

[98] Nava MM Miroshnikova YA Biggs LC Whitefield DB Metge F Boucas J . Heterochromatin-driven nuclear softening protects the genome against mechanical stress-induced damage. Cell. (2020) 181:800–817.e22. doi: 10.1016/j.cell.2020.03.052 32302590 PMC7237863

[99] Hatch EM . Nuclear envelope rupture: little holes, big openings. Curr Opin Cell Biol. (2018) 52:66–72. doi: 10.1016/j.ceb.2018.02.001 29459181 PMC5988944

[100] Resseguie EA Staversky RJ Brookes PS O’Reilly MA . Hyperoxia activates ATM independent from mitochondrial ROS and dysfunction. Redox Biol. (2015) 5:176–85. doi: 10.1016/j.redox.2015.04.012 25967673 PMC4430709

[101] Lee JU Hong J Shin H Ryu CB Park SW Jeong SH . Overexpression of V-ATPase B2 attenuates lung injury/fibrosis by stabilizing lysosomal membrane permeabilization and increasing collagen degradation. Exp Mol Med. (2022) 54:662–72. doi: 10.1038/s12276-022-00776-2 35624153 PMC9166714

[102] Li H Zhou X Tan H Hu Y Zhang L Liu S . Neutrophil extracellular traps contribute to the pathogenesis of acid-aspiration-induced ALI/ARDS. Oncotarget. (2018) 9:1772–84. doi: 10.18632/oncotarget.22744 29416730 PMC5788598

[103] Li T Chen ZJ . The cGAS-cGAMP-STING pathway connects DNA damage to inflammation, senescence, and cancer. J Exp Med. (2018) 215:1287–99. doi: 10.1084/jem.20180139 29622565 PMC5940270

[104] Storozynsky Q Hitt MM . The impact of radiation-induced DNA damage on cGAS-STING-mediated immune responses to cancer. Int J Mol Sci. (2020) 21:8877. doi: 10.3390/ijms21228877 33238631 PMC7700321

[105] Bolzán AD Bianchi MS . DNA and chromosome damage induced by bleomycin in mammalian cells: an update. Mutat Res Rev Mutat Res. (2018) 775:51–62. doi: 10.1016/j.mrrev.2018.02.003 29555029

[106] Balka KR Venkatraman R Saunders TL Shoppee A Pang ES Magill Z . Termination of STING responses is mediated via ESCRT-dependent degradation. EMBO J. (2023) 42:e112712. doi: 10.15252/embj.2022112712 37139896 PMC10267698

[107] Ergun SL Fernandez D Weiss TM Li L . STING polymer structure reveals mechanisms for activation, hyperactivation, and inhibition. Cell. (2019) 178:290–301.e10. doi: 10.1016/j.cell.2019.05.036 31230712

[108] Kattah MG Shao L Rosli YY Shimizu H Whang MI Advincula R . A20 and ABIN-1 synergistically preserve intestinal epithelial cell survival. J Exp Med. (2018) 215:1839–52. doi: 10.1084/jem.20180198 29930103 PMC6028510

[109] Zhang L Wei N Cui Y Hong Z Liu X Wang Q . The deubiquitinase CYLD is a specific checkpoint of the STING antiviral signaling pathway. PLoS Pathog. (2018) 14:e1007435. doi: 10.1371/journal.ppat.1007435 30388174 PMC6235404

[110] François-Newton V Magno de Freitas Almeida G Payelle-Brogard B Monneron D Pichard-Garcia L Piehler J . USP18-based negative feedback control is induced by type I and type III interferons and specifically inactivates interferon α response. PLoS One. (2011) 6:e22200. doi: 10.1371/journal.pone.0022200 21779393 PMC3136508

[111] Gulen MF Samson N Keller A Schwabenland M Liu C Glück S . cGAS-STING drives ageing-related inflammation and neurodegeneration. Nature. (2023) 620:374–80. doi: 10.1038/s41586-023-06373-1 37532932 PMC10412454

[112] Neufeldt CJ Cerikan B Cortese M Frankish J Lee J-Y Plociennikowska A . SARS-CoV-2 infection induces a pro-inflammatory cytokine response through cGAS-STING and NF-κB. Commun Biol. (2022) 5:45. doi: 10.1038/s42003-021-02983-5 35022513 PMC8755718

[113] Tanaka Y Chen ZJ . STING specifies IRF3 phosphorylation by TBK1 in the cytosolic DNA signaling pathway. Sci Signaling. (2012) 5:ra20. doi: 10.1126/scisignal.2002521 22394562 PMC3549669

[114] Schmid M Fischer P Engl M Widder J Kerschbaum-Gruber S Slade D . The interplay between autophagy and cGAS-STING signaling and its implications for cancer. Front Immunol. (2024) 15:1356369. doi: 10.3389/fimmu.2024.1356369 38660307 PMC11039819

[115] Gentili M Liu B Papanastasiou M Dele-Oni D Schwartz MA Carlson RJ . ESCRT-dependent STING degradation inhibits steady-state and cGAMP-induced signalling. Nat Commun. (2023) 14:611. doi: 10.1038/s41467-023-36132-9 36739287 PMC9899276

[116] Decout A Katz JD Venkatraman S Ablasser A . The cGAS-STING pathway as a therapeutic target in inflammatory diseases. Nat Rev Immunol. (2021) 21:548–69. doi: 10.1038/s41577-021-00524-z 33833439 PMC8029610

[117] Apel F Andreeva L Knackstedt LS Streeck R Frese CK Goosmann C . The cytosolic DNA sensor cGAS recognizes neutrophil extracellular traps. Sci Signaling. (2021) 14:eaax7942. doi: 10.1126/scisignal.aax7942 33688080

[118] Liu H Dong J Xu C Ni Y Ye Z Sun Z . Acute lung injury: pathogenesis and treatment. J Transl Med. (2025) 23:926. doi: 10.1186/s12967-025-06994-2 40826415 PMC12363007

[119] Sehlmeyer K Ruwisch J Roldan N Lopez-Rodriguez E . Alveolar dynamics and beyond - the importance of surfactant protein C and cholesterol in lung homeostasis and fibrosis. Front Physiol. (2020) 11:386. doi: 10.3389/fphys.2020.00386 32431623 PMC7213507

[120] Chakraborty D Zenker S Rossaint J Hölscher A Pohlen M Zarbock A . Alarmin S100A8 activates alveolar epithelial cells in the context of acute lung injury in a TLR4-dependent manner. Front Immunol. (2017) 8:1493. doi: 10.3389/fimmu.2017.01493 29180999 PMC5693860

[121] Ju YN Li H Zhuo ZP Yang Q Gao W . Mitochondrial DNA from endothelial cells activated the cGAS-STING pathway and regulated pyroptosis in lung ischaemia reperfusion injury after lung transplantation. Immunobiology. (2025) 230:152865. doi: 10.1016/j.imbio.2024.152865 39826223

[122] Zhang C-Y Ou A-J Jin L Yang N-S-Y Deng P Guan C-X . Cadmium exposure triggers alveolar epithelial cell pyroptosis by inducing mitochondrial oxidative stress and activating the cGAS-STING pathway. Cell Commun Signal CCS. (2024) 22:566. doi: 10.1186/s12964-024-01946-7 39587603 PMC11590492

[123] de Moura Rodrigues D Lacerda-Queiroz N Couillin I Riteau N . STING targeting in lung diseases. Cells. (2022) 11:3483. doi: 10.3390/cells11213483 36359882 PMC9657237

[124] Kessler N Viehmann SF Krollmann C Mai K Kirschner KM Luksch H . Monocyte-derived macrophages aggravate pulmonary vasculitis via cGAS/STING/IFN-mediated nucleic acid sensing. J Exp Med. (2022) 219:e20220759. doi: 10.1084/jem.20220759 35997679 PMC9402992

[125] Ma R Ortiz Serrano TP Davis J Prigge AD Ridge KM . The cGAS-STING pathway: the role of self-DNA sensing in inflammatory lung disease. FASEB J Off Publ Fed Am Soc Exp Biol. (2020) 34:13156–70. doi: 10.1096/fj.202001607R 32860267 PMC8121456

[126] Xie W Patel DJ . Structure-based mechanisms of 2’3’-cGAMP intercellular transport in the cGAS-STING immune pathway. Trends Immunol. (2023) 44:450–67. doi: 10.1016/j.it.2023.04.006 37147228 PMC11824902

[127] Kasper M Barth K . Potential contribution of alveolar epithelial type I cells to pulmonary fibrosis. Biosci Rep. (2017) 37:BSR20171301. doi: 10.1042/BSR20171301 29026006 PMC5696455

[128] Jacob A Morley M Hawkins F McCauley KB Jean JC Heins H . Differentiation of human pluripotent stem cells into functional lung alveolar epithelial cells. Cell Stem Cell. (2017) 21:472–488.e10. doi: 10.1016/j.stem.2017.08.014 28965766 PMC5755620

[129] Pan J Fei CJ Hu Y Wu XY Nie L Chen J . Current understanding of the cGAS-STING signaling pathway: structure, regulatory mechanisms, and related diseases. Zool Res. (2023) 44:183–218. doi: 10.24272/j.issn.2095-8137.2022.464 36579404 PMC9841179

[130] Burman A Tanjore H Blackwell TS . Endoplasmic reticulum stress in pulmonary fibrosis. Matrix Biol J Int Soc Matrix Biol. (2018) 68-69:355–65. doi: 10.1016/j.matbio.2018.03.015 29567124 PMC6392005

[131] Wang J Li S Wang M Wang X Chen S Sun Z . STING licensing of type I dendritic cells potentiates antitumor immunity. Sci Immunol. (2024) 9:eadj3945. doi: 10.1126/sciimmunol.adj3945 38363830 PMC11822965

[132] Li Z Zheng W Liu Y Cao R Wei J Jia H . Bridging innate and adaptive tumor immunity: cGAS-STING pathway activation to potentiate immune checkpoint blockade. J Exp Clin Cancer Res CR. (2025) 44:303. doi: 10.1186/s13046-025-03555-9 41204180 PMC12595792

[133] Cocconcelli E Balestro E Turato G Fiorentù G Bazzan E Biondini D . Tertiary lymphoid structures and B-cell infiltration are IPF features with functional consequences. Front Immunol. (2024) 15:1437767. doi: 10.3389/fimmu.2024.1437767 39464888 PMC11502372

[134] Zhao L Jin S Wang S Zhang Z Wang X Chen Z . Tertiary lymphoid structures in diseases: immune mechanisms and therapeutic advances. Signal Transduction Targeted Ther. (2024) 9:225. doi: 10.1038/s41392-024-01947-5 39198425 PMC11358547

[135] Wang H Kim SJ Lei Y Wang S Wang H Huang H . Neutrophil extracellular traps in homeostasis and disease. Signal Transduction Targeted Ther. (2024) 9:235. doi: 10.1038/s41392-024-01933-x 39300084 PMC11415080

[136] de Bont CM Boelens WC Pruijn GJM . NETosis, complement, and coagulation: a triangular relationship. Cell Mol Immunol. (2019) 16:19–27. doi: 10.1038/s41423-018-0024-0 29572545 PMC6318284

[137] Wienkamp AK Erpenbeck L Rossaint J . Platelets in the NETworks interweaving inflammation and thrombosis. Front Immunol. (2022) 13:953129. doi: 10.3389/fimmu.2022.953129 35979369 PMC9376363

[138] Wu B Xu M-M Fan C Feng C-L Lu Q-K Lu H-M . STING inhibitor ameliorates LPS-induced ALI by preventing vascular endothelial cells-mediated immune cells chemotaxis and adhesion. Acta Pharmacol Sin. (2022) 43:2055–66. doi: 10.1038/s41401-021-00813-2 34907359 PMC9343420

[139] Yang Z Nicholson SE Cancio TS Cancio LC Li Y . Complement as a vital nexus of the pathobiological connectome for acute respiratory distress syndrome: an emerging therapeutic target. Front Immunol. (2023) 14:1100461. doi: 10.3389/fimmu.2023.1100461 37006238 PMC10064147

[140] Feng Y Su Q Zhu J Cui X Guo J Yang J . cGAS-STING mediates neutrophil extracellular traps-induced EMT in myositis-associated interstitial lung disease: STING as a potential therapeutic target. Int Immunopharmacol. (2025) 149:114144. doi: 10.1016/j.intimp.2025.114144 39904030

[141] He X Tolosa MF Zhang T Goru SK Ulloa Severino L Misra PS . Myofibroblast YAP/TAZ activation is a key step in organ fibrogenesis. JCI Insight. (2022) 7:e146243. doi: 10.1172/jci.insight.146243 35191398 PMC8876427

[142] Qiu M Li J Wu W Ren J Wu X . The dual role of type I interferons in bacterial infections: from immune defense to pathogenesis. mBio. (2025) 16:e0148125. doi: 10.1128/mbio.01481-25 40525851 PMC12239577

[143] Messaoud-Nacer Y Culerier E Rose S Maillet I Rouxel N Briault S . STING agonist diABZI induces PANoptosis and DNA mediated acute respiratory distress syndrome (ARDS). Cell Death Dis. (2022) 13:269. doi: 10.1038/s41419-022-04664-5 35338116 PMC8953969

[144] Major J Crotta S Llorian M McCabe TM Gad HH Priestnall SL . Type I and III interferons disrupt lung epithelial repair during recovery from viral infection - PubMed. Science. (2020) 369:712–7. doi: 10.1126/science.abc2061 32527928 PMC7292500

[145] Erickson R Huang C Allen C Ireland J Roth G Zou Z . SARS-CoV-2 infection of human lung epithelial cells induces TMPRSS-mediated acute fibrin deposition. Nat Commun. (2023) 14:6380. doi: 10.1038/s41467-023-42140-6 37821447 PMC10567911

[146] Cardinal-Fernández P Lorente JA Ballén-Barragán A Matute-Bello G . Acute respiratory distress syndrome and diffuse alveolar damage. New insights on a complex relationship. Ann Am Thorac Soc. (2017) 14:844–50. doi: 10.1513/AnnalsATS.201609-728PS 28570160

[147] Qin X Fu Z Wu L Fan Y Liu X Li M . Regulation of cGAS-STING pathway with inhalable nanozyme in acute lung injury. Biomaterials. (2026) 324:123521. doi: 10.1016/j.biomaterials.2025.123521 40554963

[148] Tian M Li F Pei H Liu X Nie H . The role of the cGAS-STING pathway in chronic pulmonary inflammatory diseases. Front Med. (2024) 11:1436091. doi: 10.3389/fmed.2024.1436091 39540037 PMC11557406

[149] Kim H Wang W Dobrescu I Lee J Martorelli J Wang S . Autophagy in the lung: guardian of homeostasis or driver of disease. Autophagy Rep. (2025) 4:2568537. doi: 10.1080/27694127.2025.2568537 41098539 PMC12520118

[150] Hansel C Jendrossek V Klein D . Cellular senescence in the lung: the central role of senescent epithelial cells. Int J Mol Sci. (2020) 21:3279. doi: 10.3390/ijms21093279 32384619 PMC7247355

[151] Munger JS Huang X Kawakatsu H Griffiths MJ Dalton SL Wu J . The integrin alpha v beta 6 binds and activates latent TGF beta 1: a mechanism for regulating pulmonary inflammation and fibrosis. Cell. (1999) 96:319–28. doi: 10.1016/s0092-8674(00)80545-0 10025398

[152] Ganzleben I Medoff BD . Mechanobiology and the extracellular matrix in pulmonary fibrosis. iScience. (2025) 28:113993. doi: 10.1016/j.isci.2025.113993 41362624 PMC12682043

[153] Li J Zhai X Sun X Cao S Yuan Q Wang J . Metabolic reprogramming of pulmonary fibrosis. Front Pharmacol. (2022) 13:1031890. doi: 10.3389/fphar.2022.1031890 36452229 PMC9702072

[154] Zhou J Xia X An X Liu D Zhao H Sun Z . New perspectives on the progression of pulmonary fibrosis: the cascade from aberrant microvascular endothelial cell activation to fibrosis. Front Med (Lausanne). (2025) 12:1639043. doi: 10.3389/fmed.2025.1639043 40917842 PMC12408307

[155] Lepelley A Martin-Niclós MJ Le Bihan M Marsh JA Uggenti C Rice GI . Mutations in COPA lead to abnormal trafficking of STING to the golgi and interferon signaling. J Exp Med. (2020) 217:e20200600. doi: 10.1084/jem.20200600 32725128 PMC7596811

[156] David C Frémond ML . Lung inflammation in STING-associated vasculopathy with onset in infancy (SAVI). Cells. (2022) 11:318. doi: 10.3390/cells11030318 35159128 PMC8834229

[157] Long G Gong R Wang Q Zhang D Huang C . Role of released mitochondrial DNA in acute lung injury. Front Immunol. (2022) 13:973089. doi: 10.3389/fimmu.2022.973089 36059472 PMC9433898

[158] Nie W Lan T Yuan X . Crystalline silica induces macrophage necrosis and causes subsequent acute pulmonary neutrophilic inflammation. Cell Biol Toxicol. (2022) 38:591–609. doi: 10.1007/s10565-021-09620-1 34170461

[159] Ma Q Lim CS . Molecular activation of NLRP3 inflammasome by particles and crystals: a continuing challenge of immunology and toxicology. Annu Rev Pharmacol Toxicol. (2024) 64:417–33. doi: 10.1146/annurev-pharmtox-031023-125300 37708431 PMC10842595

[160] Lin Q Zhang CF Guo JL Su JL Guo ZK Li HY . Involvement of NEAT1/PINK1-mediated mitophagy in chronic obstructive pulmonary disease induced by cigarette smoke or PM2.5. Ann Transl Med. (2022) 10:277. doi: 10.21037/atm-22-542 35433942 PMC9011272

[161] Zhai X Wang J Sun J Xin L . PM2.5 induces inflammatory responses via oxidative stress-mediated mitophagy in human bronchial epithelial cells. Toxicol Res. (2022) 11:195–205. doi: 10.1093/toxres/tfac001 35237424 PMC8882786

[162] Schuliga M Kanwal A Read J Blokland KEC Burgess JK Prêle CM . A cGAS-dependent response links DNA damage and senescence in alveolar epithelial cells: a potential drug target in IPF. Am J Physiol Lung Cell Mol Physiol. (2021) 321:L859–71. doi: 10.1152/ajplung.00574.2020 34524912

[163] Blest HTW Chauveau L . cGAMP the travelling messenger. Front Immunol. (2023) 14:1150705. doi: 10.3389/fimmu.2023.1150705 37287967 PMC10242147

[164] Wang Y Zhang X Wang W Zhang Y Fleishman JS Wang H . cGAS-STING targeting offers therapy choice in lung diseases. Biol Direct. (2025) 20:20. doi: 10.1186/s13062-025-00611-4 39920718 PMC11806777

[165] Zeng G Chen D Zhou R Zhao X Ye C Tao H . Combination of C-reactive protein, procalcitonin, IL-6, IL-8, and IL-10 for early diagnosis of hyperinflammatory state and organ dysfunction in pediatric sepsis. J Clin Lab Anal. (2022) 36:e24505. doi: 10.1002/jcla.24505 35622931 PMC9279984

[166] Hadjadj J Yatim N Barnabei L Corneau A Boussier J Smith N . Impaired type I interferon activity and inflammatory responses in severe COVID-19 patients. Science. (2020) 369:718–24. doi: 10.1126/science.abc6027 32661059 PMC7402632

[167] Shi J-X Li J-S Hu R Li C-H Wen Y Zheng H . Diagnostic value of sTREM-1 in bronchoalveolar lavage fluid in ICU patients with bacterial lung infections: a bivariate meta-analysis. PLoS One. (2013) 8:e65436. doi: 10.1371/journal.pone.0065436 23734253 PMC3667178

[168] Wang C Kang K Lan X Fei D Wang Q Li X . Cytokine levels in sputum, not serum, may be more helpful for indicating the damage in the lung and the prognosis of severe COVID-19 - a case series. J Infect. (2021) 83:e6–9. doi: 10.1016/j.jinf.2021.08.026 34419557 PMC8375249

[169] Wang R Liu Q You W Wang H Chen Y . A transformer-based deep learning survival prediction model and an explainable XGBoost anti-PD-1/PD-L1 outcome prediction model based on the cGAS-STING-centered pathways in hepatocellular carcinoma. Brief Bioinform. (2024) 26:bbae686. doi: 10.1093/bib/bbae686 39749665 PMC11695900

[170] Dai Z Gu Z Shen R Wang J . An immunotherapy guide constructed by cGAS-STING signature for breast cancer and the biofunction validation of the pivotal gene HOXC13 via *in vitro* experiments. Front Immunol. (2025) 16. doi: 10.3389/fimmu.2025.1586877 40861477 PMC12370726

[171] Chang W Altman MD Lesburg CA Perera SA Piesvaux JA Schroeder GK . Discovery of MK-1454: a potent cyclic dinucleotide stimulator of interferon genes agonist for the treatment of cancer. J Med Chem. (2022) 65:5675–89. doi: 10.1021/acs.jmedchem.1c02197 35332774

[172] Ji N Wang M Tan C . Liposomal delivery of MIW815 (ADU-S100) for potentiated STING activation. Pharmaceutics. (2023) 15:638. doi: 10.3390/pharmaceutics15020638 36839960 PMC9966736

[173] Li M Ferretti M Ying B Descamps H Lee E Dittmar M . Pharmacological activation of STING blocks SARS-CoV-2 infection. Sci Immunol. (2021) 6:eabi9007. doi: 10.1126/sciimmunol.abi9007 34010142 PMC10021026

[174] Dorhoi A Yeremeev V Nouailles G Weiner J Jörg S Heinemann E . Type I IFN signaling triggers immunopathology in tuberculosis-susceptible mice by modulating lung phagocyte dynamics. Eur J Immunol. (2014) 44:2380–93. doi: 10.1002/eji.201344219 24782112 PMC4298793

[175] Shepardson KM Larson K Morton RV Prigge JR Schmidt EE Huber VC . Differential type I interferon signaling is a master regulator of susceptibility to postinfluenza bacterial superinfection. mBio. (2016) 7:e00506–16. doi: 10.1128/mBio.00506-16 27143388 PMC4959663

[176] Wang M Sooreshjani MA Mikek C Opoku-Temeng C Sintim HO . Suramin potently inhibits cGAMP synthase, cGAS, in THP1 cells to modulate IFN-β levels. Future Med Chem. (2018) 10:1301–17. doi: 10.4155/fmc-2017-0322 29558821

[177] Wiser C Kim B Vincent J Ascano M . Small molecule inhibition of human cGAS reduces total cGAMP output and cytokine expression in cells. Sci Rep. (2020) 10:7604. doi: 10.1038/s41598-020-64348-y 32371942 PMC7200739

[178] Wang F Ting C Riemondy KA Douglas M Foster K Patel N . Regulation of epithelial transitional states in murine and human pulmonary fibrosis. J Clin Invest. (2023) 133:e165612. doi: 10.1172/JCI16561237768734 37768734 PMC10645382

[179] Li S Hong Z Wang Z Li F Mei J Huang L . The cyclopeptide astin C specifically inhibits the innate immune CDN sensor STING. Cell Rep. (2018) 25:3405–3421.e7. doi: 10.1016/j.celrep.2018.11.097 30566866

[180] Zhao B Vo HQ Johnston FH Negishi K . Air pollution and telomere length: a systematic review of 12,058 subjects. Cardiovasc Diagn Ther. (2018) 8:480–92. doi: 10.21037/cdt.2018.06.0530214863 30214863 PMC6129826

[181] Jiménez-Loygorri JI Villarejo-Zori B Viedma-Poyatos Á Zapata-Muñoz J Benítez-Fernández R Frutos-Lisón MD . Mitophagy curtails cytosolic mtDNA-dependent activation of cGAS/STING inflammation during aging. Nat Commun. (2024) 15:830. doi: 10.1038/s41467-024-45044-1 38280852 PMC10821893

[182] Clark K Plater L Peggie M Cohen P . Use of the pharmacological inhibitor BX795 to study the regulation and physiological roles of TBK1 and IkappaB kinase epsilon: a distinct upstream kinase mediates ser-172 phosphorylation and activation. J Biol Chem. (2009) 284:14136–46. doi: 10.1074/jbc.M109.000414 19307177 PMC2682862

[183] Kraft BD Pavlisko EN Roggli VL Piantadosi CA Suliman HB . Alveolar mitochondrial quality control during acute respiratory distress syndrome. Lab Investig J Tech Methods Pathol. (2023) 103:100197. doi: 10.1016/j.labinv.2023.100197 37307952 PMC10257518

[184] Wei K Chen T Fang H Shen X Tang Z Zhao J . Mitochondrial DNA release via the mitochondrial permeability transition pore activates the cGAS-STING pathway, exacerbating inflammation in acute kawasaki disease. Cell Commun Signal CCS. (2024) 22:328. doi: 10.1186/s12964-024-01677-9 38872145 PMC11177463

[185] Mukherjee S Dasgupta S Mishra PK Chaudhury K . Air pollution-induced epigenetic changes: disease development and a possible link with hypersensitivity pneumonitis. Environ Sci pollut Res Int. (2021) 28:55981–6002. doi: 10.1007/s11356-021-16056-x34498177 34498177 PMC8425320

[186] Grazioli S Dunn-Siegrist I Pauchard LA Blot M Charles PE Pugin J . Mitochondrial alarmins are tissue mediators of ventilator-induced lung injury and ARDS. PLoS One. (2019) 14:e0225468. doi: 10.1371/journal.pone.022546831756204 31756204 PMC6874419

[187] Yang C Han Z Zhan W Wang Y Feng J . Predictive investigation of idiopathic pulmonary fibrosis subtypes based on cellular senescence-related genes for disease treatment and management. Front Genet. (2023) 14:1157258. doi: 10.3389/fgene.2023.115725837035748 37035748 PMC10079953

[188] Wobma H Shin DS Chou J Dedeoğlu F . Dysregulation of the cGAS-STING pathway in monogenic autoinflammation and lupus. Front Immunol. (2022) 13:905109. doi: 10.3389/fimmu.2022.90510935693769 35693769 PMC9186411

